# Interventions to increase the uptake of cervical cancer screening in low- and middle-income countries: a systematic review and meta-analysis

**DOI:** 10.1186/s12905-023-02265-8

**Published:** 2023-03-23

**Authors:** Khaing Nwe Tin, Chetta Ngamjarus, Siwanon Rattanakanokchai, Jen Sothornwit, Apiwat Aue-aungkul, Aye Kyawt Paing, Porjai Pattanittum, Nampet Jampathong, Pisake Lumbiganon

**Affiliations:** 1 Maternal and Reproductive Health Division, Department of Public Health, Ministry of Health, Naypyidaw, Myanmar; 2 Department of Epidemiology and Biostatistics, Faculty of Public Health, Khon Kaen University, Khon Kaen, Thailand; 3 Department of Obstetrics and Gynecology, Faculty of Medicine, Khon Kaen University, Khon Kaen, Thailand; 4 Independent Researcher, Yangon, Myanmar; 5 Cochrane Thailand, Khon Kaen University, Khon Kaen, Thailand

**Keywords:** Uptake, Cervical cancer screening, Low- and middle-income countries, Systematic review

## Abstract

**Background:**

To identify effective interventions to increase the uptake of cervical cancer screening (CCS) for low-and middle-income countries (LMICs).

**Methods:**

We searched PubMed, CENTRAL, ISI Web of Sciences, Scopus, OVID (Medline), CINAHL, LILACS, CNKI and OpenGrey for randomized controlled trials (RCTs) and cluster RCTs conducted in LMICs from January 2000 to September 2021. Two reviewers independently screened studies, extracted data, assessed risk of bias and certainty of evidence. Meta-analyses with random-effects models were conducted for data synthesis.

**Results:**

We included 38 reports of 24 studies involving 318,423 participants from 15 RCTs and nine cluster RCTs. Single interventions may increase uptake of CCS when compared with control (RR 1.47, 95% CI 1.19 to 1.82). Self-sampling of Human Papillomavirus (HPV) testing may increase uptake of CCS relative to routine Visual Inspection with Acetic Acid (RR 1.93, 95% CI 1.66 to 2.25). Reminding with phone call may increase uptake of CCS than letter (RR 1.72, 95% CI 1.27 to 2.32) and SMS (RR 1.59, 95% CI 1.19 to 2.13). Sending 15 health messages may increase uptake of CCS relative to one SMS (RR 2.75, 95% CI 1.46 to 5.19). Free subsidized cost may increase uptake of CCS slightly than $0.66 subsidized cost (RR 1.60, 95% CI 1.10 to 2.33). Community based HPV test may increase uptake of CCS slightly in compared to hospital collected HPV (RR 1.67, 95% CI 1.53 to 1.82). The evidence is very uncertain about the effect of combined interventions on CCS uptake relative to single intervention (RR 2.20, 95% CI 1.54 to 3.14).

**Conclusions:**

Single interventions including reminding with phone call, SMS, community self-sampling of HPV test, and free subsidized services may enhance CCS uptake. Combined interventions, including health education interventions and SMS plus e-voucher, may be better than single intervention. Due to low-certainty evidences, these findings should be applied cautiously.

**Supplementary Information:**

The online version contains supplementary material available at 10.1186/s12905-023-02265-8.

## Background

Although cervical cancer is a largely preventable disease, it is one of the leading causes of cancer death of women worldwide [1]. According to the Global Cancer Observatory (GLOBOCAN) 2020, cervical cancer is the fourth commonest cancer both in incidence and mortality in the world with an estimated 604,127 new cases in 2020, representing 6.5% of all female cancers. Annually, 311,365 women worldwide died from cervical cancer, which is equivalent to three deaths every five minutes. Approximately 90% of these deaths occurred in low- and middle-income countries (LMICs) [2].

Almost all of these deaths could be prevented through universal access to comprehensive cervical cancer prevention and control programs: human papillomavirus (HPV) immunization for all girls aged 9 to 13; cervical cancer screening and treatment of pre-cancerous lesions for all women with effective health education and community mobilization; and treatment of invasive cancer [1, 3]. Immunization alone could achieve the cervical cancer elimination target; however, the additional of twice-lifetime screening could accelerate the elimination by 11–31 years [4].

The majority of cervical cancer can be avoided by screening all women in the target age group for precancerous cervical lesions and treating those with positive test appropriately. Unfortunately, there are significant differences between the uptake of cervical cancer screening (CCS) among the countries [1, 3]. While the rates of CCS in high income countries (HICs) were high—more than 70% in the United Kingdom (2019) [5] and 83% in the United States (2015) [6] —those rates in LMICs were very low—20.9% in Ethiopia (2017) [7] and less than 1% in Myanmar (2018) [8].

In May 2018, the Director-General of the WHO announced a global call to action towards the elimination of cervical cancer. To eliminate cervical cancer as a public health problem globally, all countries must work towards an incidence below 4 per 100,000 women. Therefore, WHO developed and launched a global strategy to eliminate cervical cancer in November 2020, with interim targets (90–70-90) for the period 2020–2030. These targets include 90% coverage of HPV vaccination, 70% coverage of CCS, 90% coverage of the management of pre-cancers and treatment of cervical cancers [3].

One of the major challenges to having an effective CCS is the uptake of the services by women. Given the complexities of the diverse contexts and the various reasons why women did not uptake the screening service, it is difficult to analyze the situation and to determine the most effective interventions. Numerous interventions are designed to increase the uptake of CCS services. On the basis of the targeted population, these interventions can be divided into three groups: health providers, community, and women.

Various single intervention or combined interventions targeted to both health providers and community could encourage women to undergo CCS. Outreach visits, community mobilization, health education and counseling are potential components of an effective cervical cancer prevention and control program to ensure high screening coverage. Outreach strategies must reach and engage different stakeholders and community including women. Community mobilization and health education are crucial to increase awareness/knowledge and to overcome the challenges that impede access to and uptake of screening services. Counseling services are more effective to raise the women perception and utilization of the services but needs resources, private room, skilled health personnel and time. Training to health care providers could improve the interpersonal communication with community, sharing the important key message and encourage utilization the services [1].In addition, the interventions which were mainly applicable in HICs like invitation, reminding with calls or text message were somehow effective through arouse their memories and encouragement their motivation [9]. Self-sampling could also help to remove potential barriers for women participating in screening programs, such as fear of discomfort during pelvic examination, shyness and concerns with privacy. However, depends on the different interventions in different contexts, the effectiveness to increase the women’s uptake of the screening services may vary [9, 10]. Studies conducted in LMICs also revealed that there were different interventions with varying effect sizes. For instant, counseling with motivational interview in Iran [11], reminding with phone call in Malaysia [12] and self-sampling HPV testing in Ethiopia [13] showed the promising effects to increase uptake of cervical screening in LMICs.

Although the establishment of systematic, functioning, and effective screening program is essential for cervical cancer control program, it is also tremendously important to increase the uptake the screening services by women where services are available. Consequently, there is a need to identify the effective interventions to increase the uptake of screening services especially for LMICs where the majority of disease burden is greatest. A Cochrane review was conducted to evaluate all the interventions targeted at women who were eligible for screening such as invitation, reminders, education, message framing, counseling, risk factor assessment, procedures and economic [9]. Non-Cochrane reviews were also conducted to identify the effects of cervical cancer education and provider’s recommendation [10]; the nurse led interventions for all cancer early detection uptake rate [14], and the interventions to improve the cervical cancer screening uptake amongst young women [15]. These reviews, however, focused mainly on interventions which were used in HICs; therefore, the findings may not be applicable to LMICs. Also, other Cochrane reviews for interventions targeted at health professionals were the effects on professional practices and health care outcomes, not for the effects to increase the uptake of CCS [16, 17]. Moreover, there was no systematic review of the interventions to increase uptake of CCS targeted at both health care providers and women in LMICs. Therefore, this systematic review was conducted to evaluate the effectiveness and harms of the interventions targeted at health personnel, women or communities to increase the uptake of CCS in LMICs.

## Methodology

This systematic review has been registered in the Prospective Register of Systematic Reviews (PROSPERO) (https://www.crd.york.ac.uk/prospero) with the registration number of CRD42020184354.

### Criteria for considering studies for this review

#### Types of studies

We included randomized controlled trials (RCTs) and cluster randomized controlled trials conducted in LMICs based on current World Bank classifications [18], as well as studies published in all languages from 1^st^ Jan 2000 to 30^th^ September 2021 since HPV DNA test for public health use as well as population based cervical cancer screening program in LMICs were common after 2000 [19, 20], and the results of the studies in last 20 years ago might not be relevant for current practice.

#### Types of participants

Any health care providers or women who have not recently tested for cervical cancer screening or have not been diagnosed with positive screening test and cervical cancer or community in LMICs were included.

#### Type of interventions

Any single or combined interventions to motivate health care providers, women, and communities for increasing the uptake of CCS were considered. The standard/routine care or control groups were used as defined by authors. We grouped the interventions based on single intervention or combined intervention; in which more than one intervention was intervened together. Then, we classified as sub-group according to the type of interventions such as counseling, health education, reminding, invitation, messaging, economic intervention (e.g. subsidized cost), and procedures (e.g. HPV test, Visual Inspection with acetic acid (VIA) test, etc.)

#### Types of outcome measures

Primary outcomes are uptake of cervical cancer screening services and harms (e.g. social stigma, stress or anxiety, unnecessary interventions). Secondary outcomes consist of willingness to get cervical cancer screening, knowledge of screening, attitudes toward screening, satisfaction with screening service, and cost effectiveness of the interventions.

### Search methods for identification of studies

We performed searching for potential included studies from both electronic databases and other resources from 1^st^ Jan 2000 to 30^th^ September 2021. A search strategy based on the criteria for considering included studies was developed and conducted searching for PubMed, Central, ISI Web of Sciences, Scopus, OVID (Medline), CINAHL, LILACS, China National Knowledge Infrastructure (CNKI) and Open Grey (Supplementary Table S1). For databases other than MEDLINE, we adapted the search strategy accordingly. The references of included studies and any relevant systematic reviews were checked for further references to relevant trials.

### Data collection and analysis

#### Selection of studies

All the titles and abstracts retrieved from electronic database searches were imported to Mendeley software. Then, search results were merged and identified duplicates. After removing the duplicates, the remaining titles and abstracts were screened independently by two reviewers using Covidence [21] and Rayyan [22]. Then, the full text of potentially relevant studies was reviewed independently by two reviewers using the pre-specified eligibility criteria. Any discrepancies between two reviewers were resolved through discussion, however, if there were still disagreements, the third reviewer was asked to make a consensus. PRISMA flow diagram was used to illustrate the study selection process [23]. References of all included, excluded, ongoing and awaiting classification studies were presented in the appendix (Supplementary Tables S2, S3, and S4 respectively).

#### Data extraction and management

Data were extracted independently by two reviewers onto a data abstraction form specially designed for the review. Differences between reviewers were resolved by discussion and invited the third reviewer to resolve the disagreements when needed. For included trials, the following information were extracted: author; year of publication and language; country; setting; inclusion and exclusion criteria; study design; methodology; study population; number of intervention groups; intervention details; control details; outcomes were extracted. For dichotomous outcomes, numbers of women in each intervention who took screening and who responded for willingness as well as the number of women assessed at endpoint were extracted. For the continuous outcomes we extracted as mean and standard deviation and number of participants. For the cost effectiveness, we extracted the exact value. We made personal contact to trial authors in relevant fields of study when we needed clarification. Data extraction from multiple reports of the same study were collated and were handled following Cochrane handbook [24].

#### Assessment of risk of bias of the included studies

Two reviewers independently assessed the risk of bias of the included studies using the Cochrane Risk of Bias tool version 1.0 (ROB 1.0) for Randomized Controlled Trials and Cluster Randomized Controlled Trials [24]. Differences between two reviewers were resolved by discussion, if necessary, the third reviewer were asked to resolve the disagreements. Each study was assessed either as “low”, “unclear” or “high” risk for the domains of random sequence generation; allocation concealment; blinding of participants and personnel; blinding of outcome assessment; incomplete outcome data; selective reporting of outcomes; and other potential sources of bias as described in Cochrane Handbook (2019) [24]. For cluster-randomized trials, additional domains of recruitment bias; baseline imbalance; loss of clusters; incorrect analysis; and comparability with individually randomized trials were also assessed either as “low”, “unclear” or “high” risk [24].

#### Data analysis

### Measures of treatment effect

For dichotomous outcomes, we estimated a risk ratio (RR) with their 95% confidence intervals (CIs) for both individual and cluster randomized studies. For continuous outcomes, we identified two outcomes: willingness score and knowledge score. Of these two outcomes, we estimated mean difference (MD) when the outcomes from different studies measured with same scale and standardized mean difference (SMD) when the outcomes from different studies were measured with different scales, along with 95% CIs.

### Assessment of heterogeneity

Forest plot was used for visual exploration of variation between studies results. Chi-squared test and I^2^ statistic were used to determine the statistical heterogeneity among the results of included studies. We regarded as substantial heterogeneity if the p-value from Chi-squared test was less than 0.1 and the I^2^ statistic was greater than 50% [25].

### Data synthesis

We performed meta-analysis using a random-effects model to combine treatment effects from included studies. We did not combine treatment effect between included studies if the type of intervention and control were different across trials. To avoid double counting in pool estimates for the trials with more than two intervention arms, the number of events and total participants from same control or comparator group were divided for each comparison.

For cluster randomized trials, unit of analysis was cluster instead of individuals. Thus, summary statistics (for dichotomous outcome: number of events with sample size of each group, for continuous outcome: sample size of each group) were adjusted using the intra-cluster correlation coefficient (ICC). ICCs were not reported in all original trials, therefore, an external estimate of the ICC with an equivalence of 0.0295 based on Hade (2010) was used to inflate the variance of the effect estimate to all included cluster RCTs [26].

All statistical analyses were done using Review Manager 5 [27]. The results from random-effects models were presented in forest plots as the average treatment effects with 95% CIs.

### Subgroup analysis

Subgroup analyses were conducted to investigate the sources of heterogeneities. We carried out subgroup analyses in terms of type of intervention, study setting (community based/hospital based), region, publication years.

#### Sensitivity analysis

We performed sensitivity analyses by excluding studies with more than two domains of high risk of bias to determine robustness of the evidence.

#### Assessment of reporting bias

Funnel plot was carried out to investigate reporting bias for the uptake of cervical cancer screening outcome for comparison 1.

#### Summary of findings and assessment of the certainty of the evidence

The overall certainty of the evidence was assessed by two independent reviewers for primary outcome and secondary outcomes including willingness to get cervical cancer screening and knowledge of screening using Grading of Recommendations, Assessment, Development and Evaluation (GRADE) Quality Assessment Checklist [28]. The level of the certainty of the evidence was graded as “high”, “moderate”, “low” and “very low”, based on study design, risk of biases, consistency of effect, imprecision, indirectness and publication bias. We defined the post-hoc criteria for adequate precision at 15% of relative risk reduction (RRR) for binary outcomes, and at 2 of minimally important difference (MID) for continuous outcomes using SMD.

### Reporting

The reporting of this systematic review is guided by the PRISMA 2020 checklist (Preferred Reporting Items for Systematic Reviews and Meta-analyses) [29]. (Supplementary Table S5).

## Results

### Results of the search

**Fig. 1:**
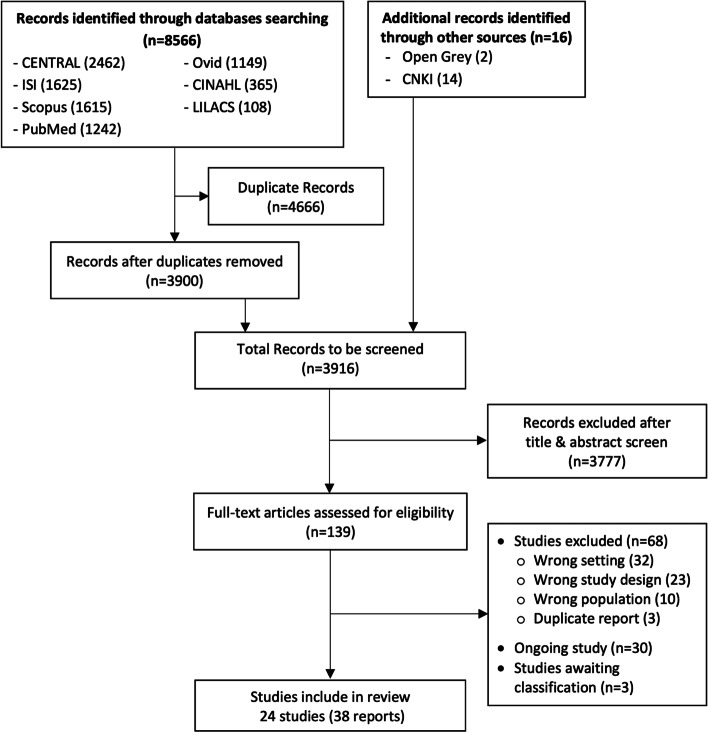
PRISMA diagram

The study selection process is displayed with PRISMA flowchart in Fig. 1. After thorough searching, 8566 reports were found from electronic database searches and 16 reports were identified from other sources. After removing the duplicates, 3916 reports were screened for title and abstracts. Of these, 3777 reports that did not meet the inclusion criteria were excluded. 139 articles were retrieved in full text to review for eligibility. After reviewing the full text, sixty-eight reports were excluded because they did not meet inclusion criteria (study setting (32 reports), study design (23 reports), population (10 reports)) and three duplicate reports. Additionally, 30 ongoing studies and 3 studies awaiting classification were not included in the review (Supplementary Table S4). Finally, 38 reports of 24 studies involving 318,423 participants were included in this review (Supplementary Table S2).

### Characteristics of included studies

All 24 studies were published in peer-reviewed journals except one study, which was found as an abstract on a website [30]. The full text of two included studies could not be obtained [30, 31] (Supplementary Table 3). Only three studies were published between 2000 to 2010 [32–34], while the rest were published between 2011 to 2021.

### Setting

Among the 24 included studies, three studies each were from Iran, Malaysia, Nigeria and Turkey; two studies each were from Ethiopia, India, Kenya, South Africa and Uganda; one study each from Cameroon and Tanzania.

### Study design

Regarding the study design, 15 studies were RCTs and nine studies were cluster RCTs. 10 studies were conducted at community-based setting including school, health insurance organization and training center; nine studies were conducted in hospital or clinic or health center; and five studies were conducted to compare the community based with hospital based setting.

### Participants

Age of the participants varied among the studies with the range of 18 to 65. There was a wide range of sample sizes across the included studies, ranging from fewer than 100 women in individual RCTs [11, 35] to 147,829 [33] and 152,010 women [34] in cluster RCTs.

### Interventions and comparisons

There were five main types of interventions. We found 13 studies used counseling or health education [11, 30, 32–42]; two studies used reminding or invitation [12, 43], three studies used messaging [44–46]; five studies used procedure (self-sampling HPV test) [13, 31, 47–49]; and one study for subsidized cost intervention [50] (Supplementary Table S6). It was remarkably noted that the interventions in all studies were targeted at the women and there was no intervention targeted at community or health providers. The details of interventions were displayed in Table 1.

**Table 1:** Characteristics of included studies

Author Year (Country)	Study design	Setting	Age of women	Type of intervention	Frequency	Duration	n
Abadi, 2018 (Iran) [11]	RCT	Healthcare/ urban centers	30–59	Counseling using motivational interview	2	1 week	45
				Routine Education	-	-	45
Abdullah, 2013 (Malaysia) [43]	Cluster RCT	School based	-	Invitation and pamphlet, follow up after 4 weeks by short phone reminder	-	10 months	199
				Standard (opportunistic) screening	-	10 months	199
Abu, 2020 (Ethiopia) [39]	Cluster RCT	Hospital based	30–49	One to one Health Education + Brochure	1	5–10 min	1062
				Standard care (no HE nor brochure)	-	-	1078
Adonis, 2017 (South Africa) [44]	RCT	Health insurance organization	21–65	Gain-framed message^a^	2	4 months	175
				Loss-framed message^b^	2	4 months	176
				Control/ Neutral-framed message^c^	2	4 months	355
Atinel, 2019 (Turkey) [30]	RCT	Primary Health Center (PHC)	40–55	Group training and delivery of brochures, training and counseling during home visits, reminder phone calls, and delivery of materials	2	40 min	67
				Routine practices	1	-	67
Dinshaw, 2008 (India) [33]	Cluster RCT	Community based	35–64	Well-planned Health Education Program (HEP) (4 rounds); VIA after 8 years	4	2 years	71,651
				Same HEP in first round and 7 rounds of active surveillance	8	1 year	76,178
Erwin, 2019 (Tanzania) [45]	RCT	Community based	25–49	SMS BCC message (15 SMS)	1	21 days	272
				SMS + e Voucher for transport	1	2 months	298
				Control (one message for location & hours of clinics)	1	-	281
Gizaw, 2020 (Ethiopia) [13]	Cluster RCT	HPV arm: Community based	30–49	Self-sampling HPV at primary health unit	1	-	1213
		VIA arm: Hospital based		VIA at hospitals	1	5 days	1143
Huchko, 2018 (Western Kenya) [47]	Cluster RCT	Community based	25–65	Community Health Clinics arm (HPV including self-collection)	3	6 weeks	2943
		Hospital based		Government health facility arm (HPV including self-collection)	-	6 weeks	3538
Koc, 2019 (Turkey) [40]	RCT	Training centers	-	Training/Health education	3	3 months	78
				Control/No training	-	-	78
Kurt, 2019 (Turkey) [36]	RCT	Community based (Home visit)	30–65	Training + education brochure	1	-	118
				Read education brochure	1	-	119
				Invitation only	-	-	119
Mitchell, 2015 (Uganda) [31]	RCT	Community based	-	Self-sampling HPV and VIA to HPV ( +)	-	-	227
		Clinic based		Standard VIA	-	-	225
Mittra, 2010 (India) [34]	ClusterRCT	Community based	35–64	Health Education and VIA	4	24 months	75,360
				Standard Health Education	1	At entry	76,178
Modibbo, 2017 (Nigeria) [48]	RCT	Community based	30–65	Self-sampling HPV	1	-	200
		Hospital based		Routine care	1	-	200
Moses, 2015 [49] (Uganda)	RCT	Community based	30–65	Self-collected HPV	1	-	250
		Hospital based		VIA	1	-	250
Okeke, 2016 (Nigeria) [50]	RCT	Community based	18–64	Subsidized cost: ₦0 (free)^d^	1	-	362
				Subsidized cost: ₦50 ($0.33)	1	-	351
				Subsidized cost: ₦100 ($0.66)	1	-	330
Okunade, 2020 (Nigeria) [46]	RCT	Hospital based	25–65	mHealth text messages containing health promotion information	12	6 months	102
				Health talk (usual care) at enrolment	1	-	98
Rashid, 2014 (Malaysia) [12]	RCT	Community clinics, suburban	20–65	Postal letter for repeated smear	-	-	250
				Registered letter	-	-	250
				SMS	-	-	250
				Phone call	-	-	250
Risi, 2004 (South Africa) [32]	RCT	Community based	35–65	Photo-comic followed by radio- drama	1	-	269
				Control-comic followed by radio- drama	1	-	389
Romli, 2020 (Malaysia) [42]	Cluster RCT	Community based	-	Standardized Health Education Program and 2 text reminders)	2	3 months	101
				Control/ 30 min health talk	1	30 min	101
Rosser, 2015 (Kenya) [41]	RCT	Hospital based	At least 23 years	30 min interactive health talk	1	30 min	207
				Control/usual care	-	-	212
Samami, 2021 (Iran) [38]	ClusterRCT	Hospital based	21–65	Educational training	1	90 min	60
				Usual care	-	-	60
Shokoohi, 2020 (Iran) [35]	Cluster RCT	Hospital based	18 years or older	Education: three one-hour training sessions	3	60 min	48
				Standard care: General information about STIs	1	-	45
Sossauer, 2014 (Cameroon) [37]	RCT	Hospital based	25–65	Educational intervention ( 6 min video promoting + ve attitude)	1	6 min	152
				Basic information	1	5 min	149

*RCT* Randomized controlled trial, *cluster RCT* cluster randomized controlled trial, *HE* Health education, *HPV* Human papillomavirus, *VIA* Visual inspection with acetic acid for cervical cancer screening, *₦* Nigerian Currency

^a^ Gained-framed message consisted of a lead-in statement that highlighted that regular Pap smears were essential to the individual’s health and wellbeing

^b^ Loss-framed message consisted of a lead-in statement that focused on the “risk” of developing cervical cancer

^c^ Neutral-framed message had no lead-in statement, only stated the recommendations for screening

^d^ Subsidized cost: 0 (free): Subsidized the service cost to free of charge to receive the cervical cancer service

In terms of control, there were standard cares or routine/usual care or no intervention such as routine health education, basic health education, standard screening, routine VIA services, and usual postal letter for reminder. In some trials, the control was defined by authors as control intervention to compare the specific interventions for that trial such as neutral-framed message and invitation only. The specific intervention which was not performed in routine care and was not classified as control by authors was not considered as control.

The following comparisons were made in this review: 1) One single intervention versus standard/ routine care/no intervention, 2) A single intervention versus a different single intervention, and 3) Combined interventions versus a single method of intervention. However, there was no comparison of 1) combined interventions versus control, and 2) combined interventions versus the other combined interventions.

### Risk of biases in included studies

**Fig. 2:**
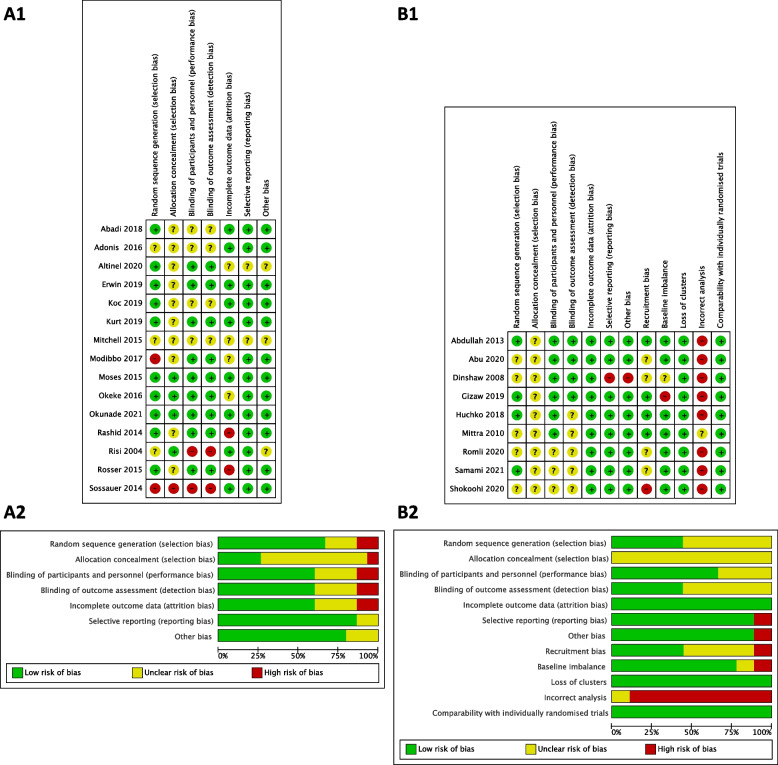
Summary of risk of bias of included studies

Figure 2 shows both risk of bias summary graphs and risk of bias graphs of included studies which were separately presented according to study design, RCTs and cluster RCTs. There was high proportion of unclear risk of bias in allocation concealment domain because most of the studies did not mention about it (Fig. 2A and B). Some unclear risk of biases was found in random sequence generation domain and two domains of blinding from RCTs and cluster RCTs. It was remarkably noted that there was high proportion of high risk of bias in incorrect analysis for cluster RCTs because clustering effects were not adjusted (Fig. 2B). Supplementary Table S7 shows the summary details for each included study.

### Effects of interventions

Among the 24 included studies, 18 studies were included in meta-analysis for the primary outcome of the uptake of CCS. Two studies did not measure the primary outcome [37, 40] and the other two studies [33, 34] measured in the intervention arm only. One study reported the change (%) in uptake with no actual numbers [30] and one study did not measure any outcomes of interest of this systematic review [35].

The secondary outcome of willingness to get CCS was reported in four studies. However, only three studies were included in data analysis because one study [34] measured the willingness only in the intervention arm. Regarding the knowledge score, five studies reported this outcome; however, one study was excluded from analysis due to its very low mean and high SD [11]. Two studies reported the attitude score; one study had very low mean and SD [11] and one study [42] reported lower score as the better attitude. Therefore, meta-analysis for attitude score could not be performed. For continuous outcome of willingness to get CCS, although two studies reported this outcome, only one study [36] with three intervention arms was included in data analysis. One study [40] was excluded from the analysis because it reported median. Knowledge as dichotomous outcome was reported in only one study [37]. Although the cost effectiveness outcome was reported in three studies, only one study with four intervention arms [12] was reviewed narratively. One study measured this outcome only in the intervention arm [51] and one study did not provide SD [52]. For the social stigma, stress, anxiety, favorable attitude, satisfaction and satisfaction score, no study reported these outcomes. In summary, a total of 19 studies were included in meta-analysis for all outcomes. The details were displayed in Supplementary Table S8.

The main effects of interventions are displayed according to the following comparisons and the rest analyses are also shown in Supplementary files.

### Comparison 1: One Single intervention versus standard/ routine care/ no intervention (control group)

#### Outcome 1: Uptake of CCS

**Fig. 3:**
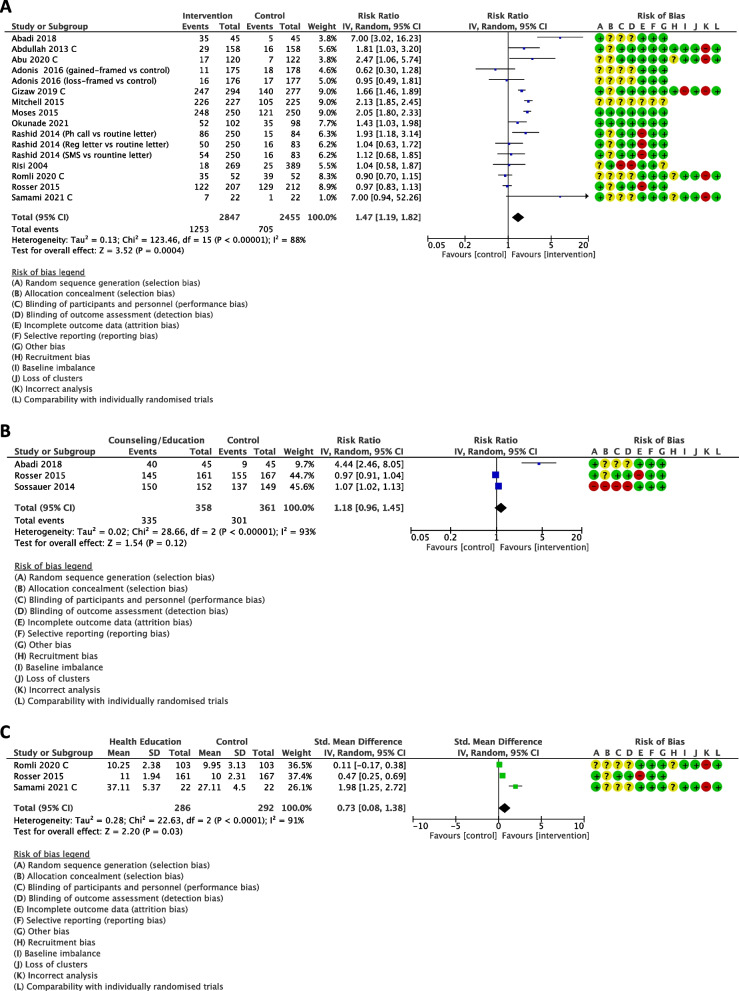
Effects of a single intervention compared with control on selected outcomes. C stands for Cluster RCTs; sample sizes of cluster RCTs are corrected with design effect estimated from ICC = 0.0295

A single intervention may increase the uptake of CCS when compared with control group (RR 1.47, 95% CI 1.19 to 1.82; 13 studies, 5302 women; low-certainty evidence, Fig. 3(A)).

Because there was substantial heterogeneity (I^2^ = 88%), subgroup analyses were conducted as presented in Supplementary Figure S1. Subgroup analysis indicated that type of intervention and study setting may make a difference to the uptake of CCS. For type of intervention, the subgroup interaction test indicated that reminding or invitation and self-sampling HPV test may increase the uptake of CCS, (RR 1.41, 95% CI 1.03 to 1.93; 2 studies, 1316 women; I^2^ = 34%; and RR 1.93, 95% CI 1.66 to 2.25; 3 studies, 1523 women; I^2^ = 75%), respectively,(Supplementary Figure S1 (A)). While for counselling or health education intervention and messaging may not increase this outcome. Subgroup analysis by study setting showed that hospital-based interventions may increase the uptake of CCS, (RR 2.14, 95% CI 1.16 to 3.95; 5 studies, 995 women; I^2^ = 87%), (Supplementary Figure S1 (B)). There was no marked difference for subgroup analysis by region (Supplementary Figure S1(C)). The evidence is very uncertain about the difference of effect size for subgroup analysis by publication year because there was only one study published during 2000–2010 (Supplementary Figure S1 (D)).

The funnel plot for this comparison 1 shows that it was roughly symmetric, indicating that publication bias is not a significant issue (Supplementary Fig. S2).

#### Outcome 2: Willingness to get cervical cancer screening (categorical)

A single intervention may have little to no effect on the willingness to get CCS when compared with control group (RR 1.18, 95% CI 0.96 to 1.45; 3 studies, 719 women; very low-certainty evidence, Fig. 3(B)).

We found high heterogeneity (I^2^ = 93%) between the trials contributing this analysis. Subgroup analysis by type of intervention, study setting, and publication year, however, could not be performed since all three studies used the same type of intervention, conducted in hospital based settings, and published after year 2010. Subgroup analysis by region indicated the difference of effect of single intervention of motivational counseling on willingness to get cervical cancer screening between Asia (RR 4.44, 95% CI 2.46 to 8.05; one study, 90 women) and Africa (RR 1.02, 95% CI 0.93 to 1.13; 2 studies, 629 women; I^2^ = 82) (Supplementary Fig. S3). This finding, however, is very uncertain because there was only one small study conducted in Asia [11].

#### Outcome 3: Knowledge score

A single intervention may increase knowledge score slightly when compared with control group (SMD 0.73, 95% CI 0.08 to 1.38; 3 studies, 578 women; low-certainty evidence, Fig. 3(C)). There was high heterogeneity (I^2^ = 91%) between studies that contributed data. The source of heterogeneity could not be found by subgroup analyses by study setting and region (Supplementary Figure S4). It was not possible to do a subgroup analysis by publication year and area due to the similarity of these characteristics across the trials included in this analysis.

### Comparison 2: A single intervention versus a different single intervention

We have not performed a meta-analysis for this comparison due to the difference in intervention type between the intervention group and the control group among the trials included in this comparison.

#### Outcome 1: Uptake of CCS

**Fig. 4:**
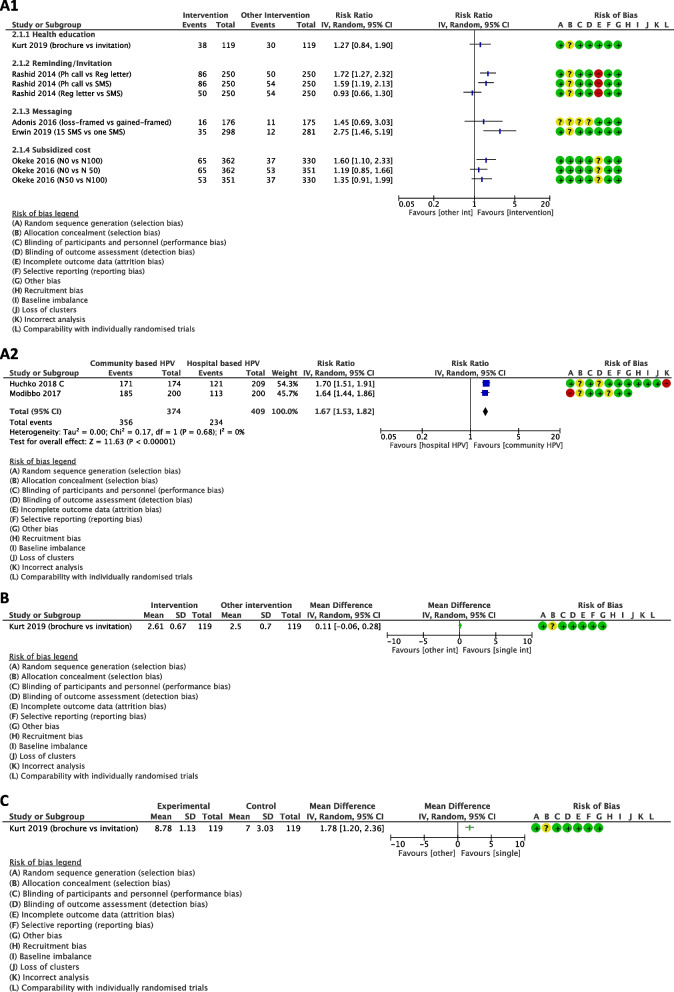
Effects of a single intervention compared with other intervention on selected outcomes. C stands for Cluster RCTs; sample sizes of cluster RCTs are corrected with design effect estimated from ICC = 0.0295

Figure 4 (A) showed the effects of single intervention on uptake of CCS when compared with a different single intervention. In terms of health education, the effect of brochure on uptake of CCS is very uncertain when compared with invitation (RR 1.27, 95% CI0.84 to 1.90; one study, 238 women; very low-certainty evidence). Reminding with phone call may increase this outcome slightly when compared with registered letter (RR 1.72, 95% CI 1.27 to 2.32; one study, 500 women; low-certainty evidence or reminding with SMS (RR 1.59, 95% CI1.19 to 2.13; one study, 500 women; low-certainty evidence). However, the effect of reminding with registered letter is very uncertain when compared with SMS (RR 0.93, 95% CI 0.66 to 1.30; one study, 500 women; very low-certainty evidence). While the difference between messaging with loss-framed and gained framed is very uncertain on this outcome (RR 1.45, 95% CI0.69 to 3.03; one study, 351 women; very low-certainty evidence), sending health education messages for 15 times may increase uptake of CCS when compared with one SMS containing clinical information (RR 2.75, 95% CI 1.46 to 5.19; one study, 579 women; low-certainty evidence). Regarding the subsidized cost, women who received ₦ 0 (free) may increase uptake of CCS slightly when compared with women who had to provide ₦ 100 ($ 0.66), RR 1.60, 95% CI 1.10 to 2.33; one study, 692 women; low-certainty evidence. On the other hand, there is little to no difference between subsidized cost ₦ 0 and ₦ 50 ($0.33) (RR 1.19, 95% CI 0.85 to 1.66; one study, 713 women; low-certainty evidence) as well as between subsidized cost ₦ 50 and ₦ 100 on this outcome (RR 1.35, 95% CI 0.91 to 1.99; one study, 681 women; low-certainty evidence). Remarkably, community-based HPV test may increase the uptake of CCS slightly when compared with hospital based HPV test, RR 1.67, 95% CI 1.53 to 1.82; 2 studies, 783 women; low-certainty evidence.

#### Outcome 2: Willingness to get cervical cancer screening (continuous)

Health education with brochure may not increase willingness to receive CCS when compared with invitation, MD 0.11, 95% CI -0.06 to 0.28; one study, 238 women; low-certainty evidence; Fig. 4(B).

#### Outcome 3: Knowledge score

Conversely, health education with brochure may increase the knowledge score for CCS slightly when compared with invitation (MD 1.78, 95% CI 1.20 to 2.36; one study, 238 women; low-certainty evidence; Fig. 4 (C)).

### Comparison 3: Combined interventions versus a single method of intervention

#### Outcome 1: Uptake of CCS

**Fig. 5:**
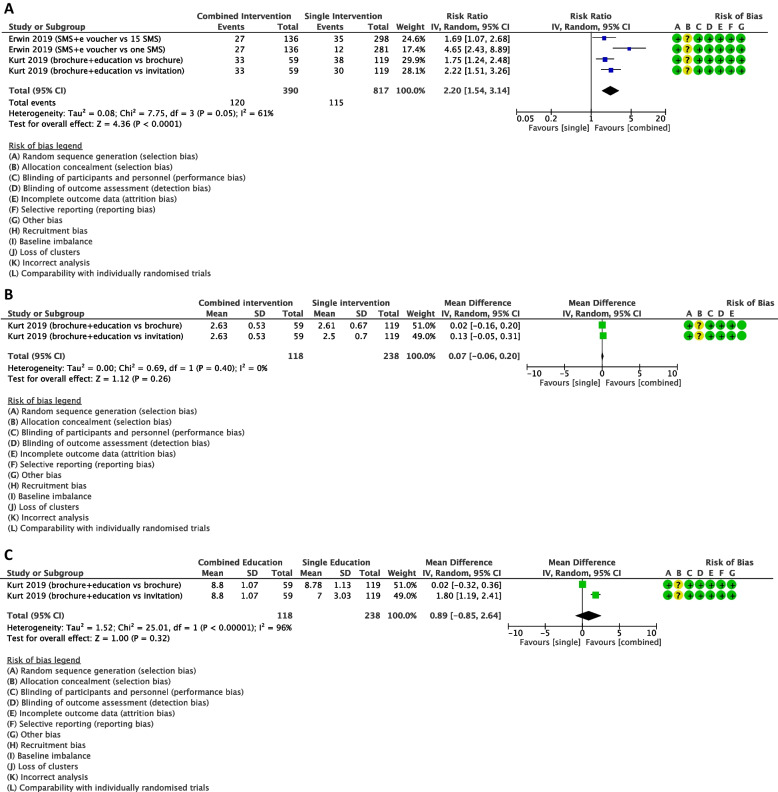
Effect of combined interventions compared with a single intervention on selected outcomes

Combined interventions may increase little to no effect on the uptake of CCS when compared to a single method of intervention but the evidence is very uncertain, RR 2.20, 95% CI 1.54 to 3.14; 2 studies, 1207 women; very low-certainty evidence; Fig. 5 (A). Due to substantial heterogeneity (I^2^ = 61%), subgroup analyses were conducted by type of intervention and by region (Supplementary Figure S5). Subgroup analysis indicated that one study of health educational intervention was conducted in Asia and one study of messaging intervention was done in Africa region. After subgroup analysis, both combined health educational interventions and combined messaging interventions may increase the uptake of screening when compared with those of single interventions (RR 1.95, 95% 1.51 to 2.52; one study, 356 women; I^2^ = 0%, Supplementary Figure S5; and RR 2.73, 95% CI 1.01 to 7.34; one study, 851 women; I^2^ = 84%; Supplementary Figure S5; respectively). Combined intervention of brochure and education may increase the uptake of CCS slightly when compared to a single method of intervention with brochure alone, (RR 1.75, 95% CI 1.24 to 2.48; one study, 178 women; Fig. 5 (A)) or invitation alone, (RR 2.22, 95% CI 1.51 to 3.26; one study, 178 women; Fig. 5 (A)). In addition, SMS plus e-voucher for transport to screen may increase the uptake of CCS when compared to sending 15 SMS, (RR 1.69, 95%CI 1.07 to 2.68; one study, 434 women; Fig. 5 (A)) or one SMS, (RR 4.65, 95% CI 2.43 to 8.89; one study, 417 women; Fig. 5 (A)).

#### Outcome 2: Willingness to get CCS (continuous outcome)

Combined intervention may not increase the willingness to get CCS when compared with a single method of intervention, MD 0.07, 95% CI -0.06 to 0.20; one study, 356 women; low-certainty evidence; Fig. 5 (B).

#### Outcome 3: Knowledge score

Combined health educational interventions may have little to no effect on the knowledge score when compared with a single intervention, MD 0.89, 95% CI -0.85 to 2.64; one study, 356 women; very low-certainty evidence; Fig. 5 (C). However, combined brochure plus health education may increase knowledge score slightly when compared with invitation letter, MD 1.80, 95% CI 1.19 to 2.41; one study, 178 women; Fig. 5 (C).

### Other outcomes those are not included in comparisons

One trial; with four domains of high risk of bias, revealed that educational intervention may increase the proportion of satisfactory knowledge group over the control [37]. However, we did not identify any effective intervention to increase the attitude score on cervical cancer screening. Reminding with phone call, HPV screen and treat method and screening at Community Health Campaigns were cost effective strategy to increase uptake of CCS [12, 51, 52]. The details are mentioned in Supplementary 3.

### Sensitivity analysis

We identified two studies with more than two high-risk domains of bias [33, 37], however Dinshaw 2008 [33]was not included in any meta-analysis because this study reported only the results of the intervention arm. For Sossauer 2014 [37], this trial was included in meta-analysis of comparison 1 for the willingness to get cervical cancer screening outcome. The sensitivity analysis reveals no significant difference in the magnitude of the effect after excluding this trial (Supplementary Figure S6).

## Discussion

### Summary of main results

#### A Single intervention versus standard/ routine care/ no intervention (control group)

When a single intervention compared with the control group, the evidence suggests that a single intervention may increase the uptake of CCS. However, there was substantial heterogeneity for this outcome. Subgroup analysis suggested a potential difference by type of intervention and study setting. Where using reminding or invitation, self-sampling HPV test as well as hospital-based interventions may increase the uptake of CCS. In addition, a single intervention may increase knowledge score slightly relative to the control group. This outcome had a high level of heterogeneity; unfortunately, subgroup analyses could not explore source of heterogeneity. This result should therefore be interpreted with caution. In comparison to the control group, a single intervention may have little to no effect on the willingness to get cervical cancer screening. High heterogeneity was found for this outcome, which revealed that a single intervention may enhance the willingness to undergo cervical cancer screening in one Asian trial.

#### A single intervention versus a different single intervention

A single intervention of reminding with phone call may increase the uptake of CCS slightly when compared with a different single intervention such as registered letter or SMS. In comparison to one SMS, sending 15 health education messages may increase uptake of CCS. While provision of cervical cancer screening service with free subsidized cost (₦ 0) may increase uptake of CCS slightly relative to ₦ 100 ($0.66), the difference is uncertain between ₦ 0 and ₦ 50 ($0.33) as well as between ₦ 50 and ₦ 100. Nevertheless, community based self-sampling of HPV test may increase the uptake of CCS slightly in compared to hospital based HPV test. When compared with invitation, education with brochure may increase the knowledge score; however, it may not increase the willingness to get screening.

#### Combined interventions versus a single method of intervention

In comparison to a single method of intervention, combined interventions may increase little to no effect on the uptake of CCS; though there was substantial heterogeneity. Subgroup analysis suggested combined health educational interventions as well as combined messaging interventions may increase the uptake of CCS when compared to a single method of those interventions. Also, combined intervention of brochure and education may increase the uptake of CCS slightly when compared to a single method of intervention of either brochure or invitation alone. In addition, combined intervention of SMS plus e-voucher for transport to screen may increase the uptake of CCS than either sending 15 SMS containing health information or sending one SMS of clinic information. However, the difference between combined interventions and single intervention is little or no effect on both willingness to get screening and knowledge score. It was remarkably observed that combined brochure plus health education may increase knowledge score slightly relative to invitation.

### Overall completeness and applicability of evidence

To our knowledge, this is the first systematic review for all the interventions targeted to either women or health providers to increase the uptake of CCS which restricted to LMICs. Our review provides the additional insights into the results of existing review especially for LMICs. Although this review is limited to LMICs, only 11 countries from two continents (Asia and Africa) were found. All the interventions from included trials are targeted to women and could not identify the trials targeted to health professional or community. Nevertheless, all the possible age range of women (18 to 65) was included in this review. Due to limited numbers of included studies for the subsidized cost intervention; where only one study with three intervention arms, and combined interventions of health education, the results should be considered carefully. In addition to primary outcomes, the other secondary outcomes such as willingness, knowledge, attitude, and cost effectiveness are also reported. However, this review could not assess the harms of the interventions on cervical cancer screening due to no trials reported any harms such as social stigma, stress or anxiety, unnecessary interventions. Most of the included studies were published after 2010. Only RCTs and cluster RCTs were included, and meta-analyses were done to estimate the pooled results. The sample sizes for cluster RCTs are also adjusted with the most reliable imputed ICC even though the included studies were not corrected for clustering effects. Sensitivity analyses excluding trials with more than two domains of high risk of bias were conducted to check the robustness of the results.

### Certainty of the evidence

**Fig. 6:**
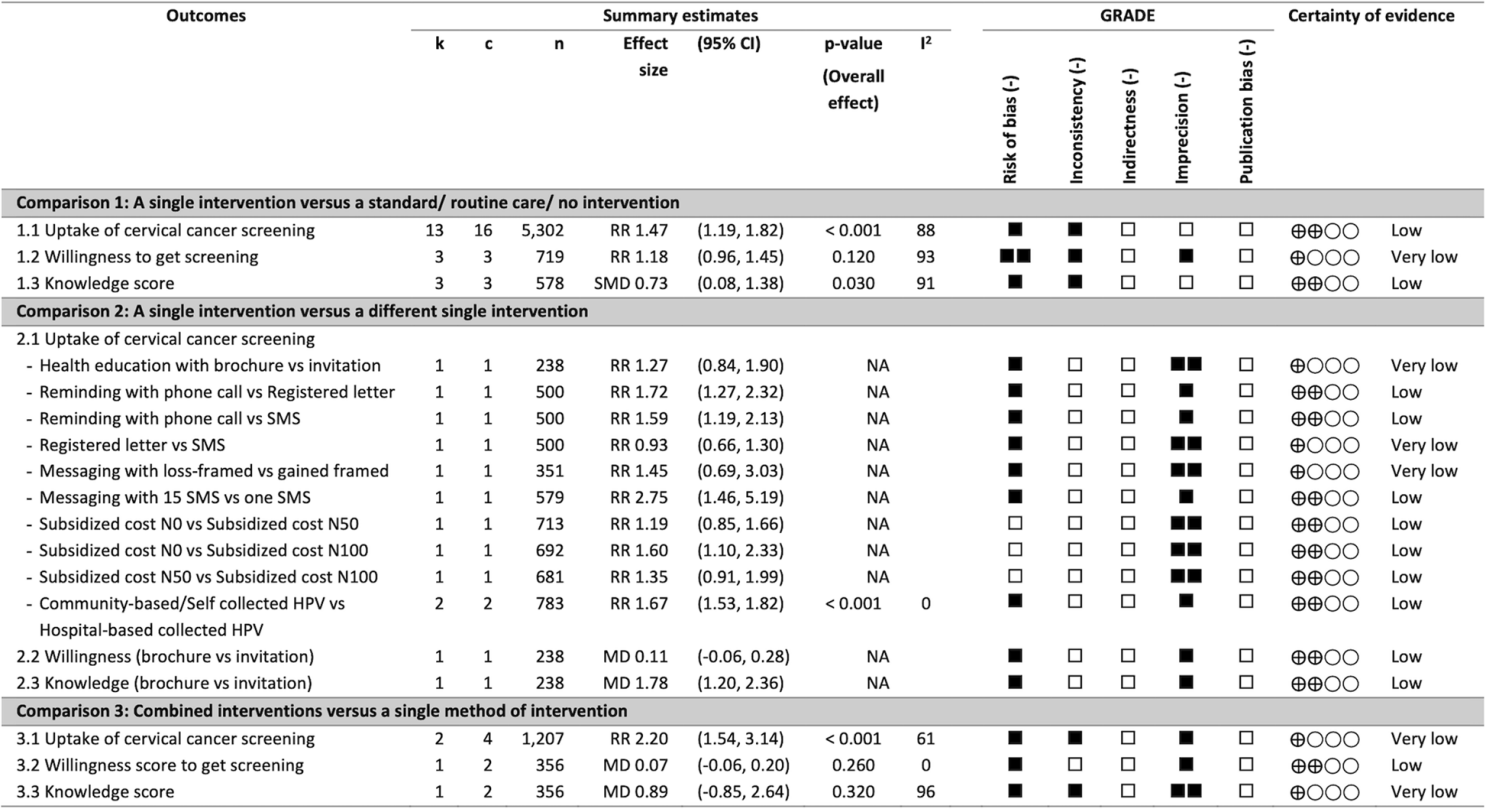
Summary of findings table for primary outcomes. ■  = downgrade one level; □ = not downgrade one level for each GRADE domain; ⨁ = level of certainty of evidence (see full details in Supplementary Tables S9 to S11); k = number of studies; c = number of a pair-wise comparison of interventions (multi-arm study); *n* = number of participants; RR = risk ratio; MD = mean difference; SMD = standardized mean difference; CI = confidence interval

In this review, the certainty of evidence ranges from low to very low. The details are shown in the GRADE/ Summary of findings tables (Fig. 6, Supplementary Tables 9, 10 and 11). For risk of bias, a great proportion of trial methods were not well reported; therefore, it was downgraded one level for risk of bias in most of the studies when there was an unclear risk of bias in allocation of concealment alone or both domains of selection bias (random sequence generation and allocation concealment). When there were high risk of biases for four domains in one study (Sossauer 2014), included in comparison 1 of willingness outcome, we downgraded two levels. We also downgraded one level for serious inconsistency due to substantial heterogeneity. There is no study for serious indirectness. We downgraded one level for serious imprecision if there was a few event rate and/or few sample size and, further downgraded one more level if there was a wide CI using the criteria of 15% of RRR for binary outcomes, and at 2 of MID for continuous outcomes. No serious issue was detected for publication bias.

### Potential bias in review process

To reduce the potential bias in review, data extraction and assessment of risk of bias were performed independently by two reviewers. Differences between reviewers were resolved by discussion and invited the third reviewer when needed. Restriction to include only RCTs and cluster RCTs and exclusion of quasi experimental studies and other non-RCTs were performed to get the strong evidence. For those studies reported only the results of intervention arms and low mean and high SD of attitude score, we contacted to authors but not received the reply; thus these studies were not included analysis. Assessment of funnel plot showed the publication bias was not a prominent issue for our primary outcome. However, using the results of two studies from abstracts may have some bias.

### Agreements and disagreements with other studies or reviews

Musa et.al reported that the use of theory-based educational interventions increased two times of CCS rates (OR, 2.46, 95% CI 1.88 to 3.21; 5 studies, 1609 women; I^2^ = 24%) and offering women the option of self-sampling for HPV testing also increased cervical cancer screening rates by nearly two-fold (OR, 1.71, 95% CI 1.32 to 2.22; 8 studies, 40,570 women; I^2^ = 94%) [10]. Another systematic review of the effectiveness of health education interventions indicated that health education interventions have immense contributions in boosting the screening uptake. However, the pooled effects of health education was not estimated and reported as the effectiveness are different based on the study setting, populations and the way of delivery [53]. Recently published systematic review reported that the effect of educational interventions among rural women increased the uptake of CCS (RR, 1.26, 95% CI 1.10 to 1.45; 2 studies, 663 women,; I^2^ = 9%) [54]. A narrative review conducted among lower socioeconomic group in HICs reported that HPV self-testing can improve uptake of cervical cancer screening among lower socioeconomic groups. And, it also informed that the use of lay health advisors had statistically significant increase in screening uptake [55]. A meta-analysis of the four RCTs conducted in sub-Sahara Africa showed that the uptake of women using self-sampling HPV test is 72% higher than those using the standard of care (RR 1.72, 95% CI 1.58 to 1.87; I^2^ = 72%). The substantial heterogeneity 72% could be explained by subgroup analysis of recruitment timing: immediate (RR 2.05, 95% CI 1.80 to 2.33, one study, 500 women) and with some time range (RR 1.65, 95% CI 1.58 to 1.72, 3 studies, 7700 women, I^2^ = 0%) [56]. Staley et.al updated the Cochrane review published in 2011 and described as moderate‐certainty evidence supports the use of invitation letters to increase the uptake of cervical screening. Low‐certainty evidence presented the use of lay health worker amongst ethnic minority populations may increase the screening coverage, and also supports the educational interventions although the most effective format is unclear. Also, the majority of included studies in this review were HICs and so the applicability in LMICs is uncertain [57].

## Conclusion

Low certainty evidence suggests that single interventions may increase uptake of CCS slightly when compared with control; in which reminding, invitation and self-sampling HPV test were effective. Phone call is the most effective reminding method relative to other reminding interventions such as registered letter or SMS (low-certainty evidence). Community based self-sampling of HPV test was also an effective intervention to increase the uptake of cervical cancer screening than either routine VIA services or hospital collected HPV test (low-certainty evidence). Sending 15 SMS contained health information is more effective than sending one SMS contained only clinic information (low-certainty evidence). In comparison to single intervention, combined interventions may increase little to no effect on the uptake of CCS (very low-certainty evidence); however, the evidence is uncertain. Combined health educational intervention; brochure and education, is more likely to increase the uptake than the single intervention of either invitation or brochure alone. Similarly, combined interventions of SMS plus e voucher for transport to screen may increase when compared with 15 SMS or one SMS.

However, due to low-and very low- certainty of evidence, the findings should be applied cautiously. Further RCTs are required to better understand the subsidized cost intervention; where only one study with three intervention arms was included in this review. In addition, further RCTs evaluating the effects of combined interventions are required to get the strong evidence since the number of included trials for those combined intervention is limited. Finally, further studies which target the health providers or community or evaluate the harms of the cervical cancer screening interventions are also recommended.

## Supplementary Information

**Additional file 1:** **Supplementary 1.** Differences between protocol and review. **Supplementary 2.** Design effect calculation for cluster RCTs. **Supplementary 3.** Other outcomes those are not included in comparisons. **Supplementary Figure S1.** Effect of single intervention on uptake of cervical cancer screening when compared with control according to (A) type of intervention, (B) study setting, (C) region, and (D) publication year. **Supplementary Figure S2.** Funnel plot of the effect of single intervention on uptake of cervical cancer screening when compared with control. **Supplementary Figure S3.** Effect of single intervention on willingness to get cervical cancer screening when compared with control according to region. **Supplementary Figure S4.** Effect of single intervention on knowledge score when compared with control according to (A) study setting and (B) region. **Supplementary Figure S5.** Effect of combined interventions on uptake of cervical cancer screening when compared with single intervention according to (A) type of intervention and (B) region. **Supplementary Figure S6.** Sensitivity analysis for effect of single intervention on willingness to get cervical cancer when compared with control by removing study with more than two domains of high risk of bias. **Supplementary Table S1.** Search strategy. **Supplementary Table S2.** List of included studies. **Supplementary Table S3.** List of excluded studies. **Supplementary Table S4.** List of studies ongoing and awaiting classification. **Supplementary Table S5.** PRISMA checklist. **Supplementary Table S6.** Included studies by types of interventions. **Supplementary Table S7.** Judgement for risk of bias assessment using the Cochrane Risk of Bias Tool for Randomized Controlled Trials. **Supplementary Table S8.** Summary of reported outcomes of the included studies. **Supplementary Table S9.** Summary of findings table (single intervention compared with control for uptake cervical cancer screening). **Supplementary Table S10.** Summary of findings table (single intervention compared with other intervention for uptake cervical cancer screening). **Supplementary Table S11.** Summary of findings table (combined interventions compared with single intervention for uptake cervical cancer screening).

## Data Availability

All data generated or analyzed during this study are included in this published article or the supplementary file.

## Abbreviations

CCS: Cervical cancer screening
CIs: Confidence intervals
CNKI: China national knowledge infrastructure
GLOBOCAN: Global cancer observatory
GRADE: Grading of recommendations, assessment, development and evaluation
HICs: High income countries
HPV: Human papillomavirus
ICC: Intraclass correlation coefficient
LMICs: Low- and middle- income countries
MD: Mean difference
MID: Minimally important difference
PRISMA: Preferred reporting items for systematic review and meta-analyses
PROSPERO: Prospective register of systematic reviews
RCTs: Randomized controlled trials
ROB: Risk of bias
RR: Risk ratio
RRR: Relative risk reduction
SD: Standard deviation
SMD: Standardized mean difference
VIA: Visual inspection with acetic acid
WHO: World health organization

## References

[1] World Health Organization (2014). Comprehensive cervical cancer control: a guide to essential practice.

[2] GLOBOCAN. New Global Cancer Data. 2020. https://www.uicc.org/news/globocan-2020-new-global-cancer-data. Accessed 15 Mar 2020.

[3] World Health Organization. Global strategy to accelerate the elimination of cervical cancer as a public health problem and its associated goals and targets for the period 2020 – 2030. 2020.

[4] Brisson M, Kim JJ, Canfell K, Drolet M, Gingras G, Burger EA (2020). Impact of HPV vaccination and cervical screening on cervical cancer elimination: a comparative modelling analysis in 78 low-income and lower-middle-income countries. Lancet.

[5] Cervical screening: data and research - GOV.UK. https://www.gov.uk/government/collections/cervical-screening-programme-data#cervical-screening-coverage. Accessed 15 Mar 2020.

[6] American Cancer Society. Cancer Prevention & Early Detection. 2015. https://www.cancer.org/research/cancer-facts-statistics/cancer-prevention-early-detection.html. Accessed 15 Mar 2020.

[7] AnimenBante S, Getie SA, Getu AA, Mulatu K, Fenta SL (2017). Uptake of pre-cervical cancer screening and associated factors among reproductive age women in Debre Markos town. Northwest Ethiopia.

[8] Maternal and Reproductive Health Division. Guideline on Secondary Prevention of Cervical Cancer (Myanmar). Ministry of Health and Sports, Myanmar.

[9] Everett T, Bryant A, Griffin MF, Martin-Hirsch PPL, Forbes CAJR (2011). Interventions targeted at women to encourage the uptake of cervical screening (Review). Cochrane Database Syst Rev.

[10] Musa J, Achenbach CJ, O’dwyer LC, Evans CT, Mchugh M, Hou L (2017). Effect of cervical cancer education and provider recommendation for screening on screening rates: A systematic review and meta-analysis. PloS one.

[11] Abadi MMM, Vakilian K, Safari V (2018). Motivational interview on having pap test among middle-aged women – A counseling service in primary care. Fam Med Prim Care Rev.

[12] Rashid RMA, Ramli S, John J, Dahlui M (2014). Cost Effective analysis of recall methods for cervical cancer screening in selangor - results from a prospective randomized controlled trial. Asian Pac J Cancer Prev.

[13] Gizaw M, Ruddies F, Addissie A, Worku A, Abebe T, Teka B (2020). Abstract B103: Community-based uptake of self-sampling for HPV DNA-based testing for cervical cancer screening in Ethiopia: Preliminary findings of a cluster randomized trial. Cancer Epidemiol Prev Biomarkers.

[14] Li C, Liu Y, Xue D, Chan CWH (2020). Effects of nurse-led interventions on early detection of cancer: A systematic review and meta-analysis. Int J Nurs Stud.

[15] Albrow R, Blomberg K, Kitchener H, Brabin L, Patnick J, Tishelman C (2014). Interventions to improve cervical cancer screening uptake amongst young women: A systematic review. Acta Oncol (Madr).

[16] Ma OB, Rogers S, Jamtvedt G, Ad O, Dt K, Forsetlund L (2007). Educational outreach visits: effects on professional practice and health care outcomes (Review). Cochrane Database Syst Rev.

[17] Arditi C, Rège-Walther M, Durieux P, Burnand B (2017). Computer-generated reminders delivered on paper to healthcare professionals: Effects on professional practice and healthcare outcomes. Cochrane Database Syst Rev.

[18] World Bank. New World Bank country classifications by income level: 2020–2021. 2020.

[19] Cox JT (2009). History of the use of HPV testing in cervical screening and in the management of abnormal cervical screening results. J Clin Virol.

[20] Sivaram S, Majumdar G, Perin D, Nessa A, Broeders M, Lynge E (2018). Population-based cancer screening programmes in low-income and middle-income countries: regional consultation of the International Cancer Screening Network in India HHS Public Access. Lancet Oncol.

[21] Covidence. Cochrane Community. https://community.cochrane.org/help/tools-and-software/covidence. Accessed 22 Mar 2022.

[22] Rayyan – Intelligent Systematic Review. https://www.rayyan.ai/. Accessed 22 Mar 2022

[23] Liberati A, Altman DG, Tetzlaff J, Mulrow C, Gøtzsche PC, Ioannidis JPA (2009). The PRISMA statement for reporting systematic reviews and meta-analyses of studies that evaluate health care interventions: Explanation and elaboration. PLoS Med.

[24] Higgins J, Savović J, Page M, Elbers R, Sterne J. Cochrane Handbook for Systematic Reviews of Interventions (Version 6.0). Cochrane; 2019.

[25] Higgins J, Green S, editors. Cochrane Handbook for Systematic Reviews of Interventions Version 5.1.0 (updated March 2011). The Cochrane Collaboration; 2011.

[26] Hade EM, Murray DM, Pennell ML, Rhoda D, Paskett ED, Champion VL (2010). Intraclass correlation estimates for cancer screening outcomes: Estimates and applications in the design of group-randomized cancer screening studies. J Natl Cancer Inst - Monogr.

[27] Review Manager (RevMan). The Cochrane Collaboration. 2020. https://revman.cochrane.org/

[28] Higgins J, Thomas J, Chandler J, Cumpston M, Li T, Page M. Chapter 14: Completing ‘Summary of findings’ tables and grading the certainty of the evidence. 2022. https://training.cochrane.org/handbook/current/chapter-14. Accessed 20 Aug 2022.

[29] Page MJ, McKenzie JE, Bossuyt PM, Boutron I, Hoffmann TC, Mulrow CD, The PRISMA (2020). statement: An updated guideline for reporting systematic reviews. BMJ.

[30] Altinel B, Akin B (2019). The effect of multiple interventions for women at risk for cervical cancer on their health responsibility, beliefs regarding cervical cancer, and having screening: A randomized controlled experiment. Ann Oncol.

[31] Mitchell S, Moses H, Seikubo M, Mwesigwa D, Singer J (2015). Examining HPV Prevalence and Uptake of Cervical Cancer Screening among HIV positive and negative women participating in a pilot RCT in Uganda comparing self-collection based HPV testing to VIA. Sex Transm Infect.

[32] Risi L, Bindman JP, Campbell OMR, Imrie J, Everett K, Bradley J (2004). Media interventions to increase cervical screening uptake in South Africa : an evaluation study of effectiveness. Health Educ Res.

[33] Dinshaw K, Mishra G, Shastri S, Badwe R, Kerkar R, Ramani S (2007). Reducing the worldwide burden of cancer determinants of compliance in a cluster randomised controlled trial on screening of breast and cervix cancer in Mumbai. India Oncol.

[34] Mittra I, Mishra GA, Singh S, Aranke S, Notani P, Badwe R (2010). cancer screening in Mumbai, India : methodology and interim results after three rounds of screening. Int J cancer.

[35] Shokoohi M, Jamshidimanesh M, Ranjbar H, Saffari M, Motamed A (2020). The effectiveness of a model-based health education program on protective behavior against human papillomavirus in female drug abusers: a randomized controlled trial. HIV AIDS Rev.

[36] Kurt G, Akyuz A (2019). Evaluating the effectiveness of interventions on increasing participation in cervical cancer screening. J Nurs Res.

[37] Sossauer G, Zbinden M, Tebeu P, Fosso K, Untiet S, Vassilakos P (2014). Impact of an educational intervention on women ’ s knowledge and acceptability of human papillomavirus self-sampling : a randomized controlled trial in. PLoS ONE.

[38] Samami E, Seyedi-Andi SJ, Bayat B, Shojaeizadeh D, Tori NA (2021). The effect of educational intervention based on the health belief model on knowledge, attitude, and function of women about Pap smear test at Iranian health centers: A randomized controlled clinical trial. J Educ Health Promot.

[39] Abu SH, Woldehanna BT, Nida ET, Tilahun W, Gebremariam MY, Sisay MM (2020). The role of health education on cervical cancer screening uptake at selected health centers in Addis Ababa. PLoS ONE.

[40] Koc Z, Ozdez EK, Topatan S (2019). The Impact of Education About Cervical Cancer and Human Papillomavirus on Women’s Healthy Lifestyle Behaviors and Beliefs. Wolters Kluwer Heal Inc.

[41] Rosser JI, Njoroge B, Huchko MJ (2015). Patient education and counseling changing knowledge , attitudes , and behaviors regarding cervical cancer screening : the effects of an educational intervention in rural Kenya. Patient Educ Couns.

[42] Romli R, Shahabudin S, Saddki N, Mokhtar N, Romli RSSSN, Mokhtar N (2020). Effectiveness of a health education program to improve knowledge and attitude towards cervical cancer and pap smear: A controlled community trial in Malaysia. Asian Pacific J Cancer Prev.

[43] Abdullah F, Rorke MO, Murray L, Su TT (2013). Evaluation of a Worksite cervical screening initiative to increase pap smear uptake in Malaysia: A Cluster randomized controlled trial. Biomed Res Int.

[44] Adonis L, Paramanund J, Basu D, Luiz J. Framing preventive care messaging and cervical cancer screening in a health-insured population in South Africa: implications for population-based communication? J Health Psychol. 2017;22(11):1365–75. 10.1177/1359105316628735 26888327

[45] Erwin E, Aronson KJ, Day A, Ginsburg O, Macheku G, Feksi A (2019). SMS behaviour change communication and eVoucher interventions to increase uptake of cervical cancer screening in the Kilimanjaro and Arusha regions of Tanzania : a randomised, double-blind, controlled trial of effectiveness. BMJ Innov.

[46] Okunade KS, Salako O, Adejimi AA, Akinsola OJ, Fatiregun O, Adenekan MA (2020). Impact of mobile technologies on cervical cancer screening practices in Lagos , Nigeria (mHealth-Cervix ): Protocol for a randomised controlled trial. F1000Research.

[47] Huchko MJ, Ibrahim S, Blat C, Cohen CR (2018). Cervical cancer screening through human papillomavirus testing in community health campaigns versus health facilities in rural western Kenya. Int J Gynaecol Obstet.

[48] Modibbo F, Iregbu KC, Okuma J, Leeman A, Kasius A, De KM (2017). Randomized trial evaluating self-sampling for HPV DNA based tests for cervical cancer screening in Nigeria. Infect Agent Cancer.

[49] Moses E, Pedersen H, Mitchell S, Sekikubo M (2015). Towards the best model for cervical cancer screening in low and middle income countries: results from a pilot randomized controlled trial comparing self-collected HPV testing with VIA in Uganda. Int J Gynecol Obstet.

[50] Okeke EN (2016). Does price affect the demand for information about new health technologies? evidence from a field experiment in Nigeria. Univ Chicago Press.

[51] Mezei AK, Pedersen HN, Sy S, Regan C, Mitchell-foster SM, Byamugisha J (2018). Community-based HPV self-collection versus visual inspection with acetic acid in Uganda : a cost-effectiveness analysis of the ASPIRE trial. BMJ Open.

[52] Shen J, Olwanda E, Kahn JG, Huchko MJ (2018). Cost of HPV screening at community health campaigns ( CHCs ) and health clinics in rural Kenya. BMC Health Serv Res.

[53] Agide FD, Garmaroudi G, Sadeghi R, Shakibazadeh E, Yaseri M, Koricha ZB (2018). A systematic review of the effectiveness of health education interventions to increase cervical cancer screening uptake. Eur J Public Health.

[54] Zhang M, Sit JWH, Chan DNS, Akingbade O, Chan CWH (2022). Educational interventions to promote cervical cancer screening among rural populations: a systematic review. Int J Environ Res Public Health.

[55] Rees I, Jones D, Chen H, Macleod U (2018). Interventions to improve the uptake of cervical cancer screening among lower socioeconomic groups: A systematic review. Prev Med (Baltim).

[56] Tesfahunei HA, Ghebreyesus MS, Assefa DG, Zeleke ED, Acam J, Joseph M (2021). Human papillomavirus self-sampling versus standard clinician-sampling for cervical cancer screening in sub-Saharan Africa: a systematic review and meta-analysis of randomized controlled trials. Infect Agents Cancer.

[57] Staley H, Shiraz A, Shreeve N, Bryant A, Martin-Hirsch PPL, Gajjar K. Interventions targeted at women to encourage the uptake of cervical screening. Cochrane Database Syst Rev. 2021;9(9):CD002834. 10.1002/14651858.CD002834.pub3 PMC8543674 34694000

