# Supplementary material for: Interventions to increase the uptake of cervical cancer screening in low- and middle-income countries: a systematic review and meta-analysis
Source: BMC Womens Health. 2023 Mar 23;23:120. doi: 10.1186/s12905-023-02265-8 (PMC10035175; doi:10.1186/s12905-023-02265-8)
Supplement: Supplementary file 1 — Additional file 1: Supplementary 1. Differences between protocol and review. Supplementary 2. Design effect calculation for cluster RCTs. Supplementary 3. Other outcomes those are not included in comparisons. Supplementary Figure S1. Effect of single intervention on uptake of cervical cancer screening when compared with control according to (A) type of intervention, (B) study setting, (C) region, and (D) publication year. Supplementary Figure S2. Funnel plot of the effect of single intervention on uptake of cervical cancer screening when compared with control. Supplementary Figure S3. Effect of single intervention on willingness to get cervical cancer screening when compared with control according to region. Supplementary Figure S4. Effect of single intervention on knowledge score when compared with control according to (A) study setting and (B) region. Supplementary Figure S5. Effect of combined interventions on uptake of cervical cancer screening when compared with single intervention according to (A) type of intervention and (B) region. Supplementary Figure S6. Sensitivity analysis for effect of single intervention on willingness to get cervical cancer when compared with control by removing study with more than two domains of high risk of bias. Supplementary Table S1. Search strategy. Supplementary Table S2. List of included studies. Supplementary Table S3. List of excluded studies. Supplementary Table S4. List of studies ongoing and awaiting classification. Supplementary Table S5. PRISMA checklist. Supplementary Table S6. Included studies by types of interventions. Supplementary Table S7. Judgement for risk of bias assessment using the Cochrane Risk of Bias Tool for Randomized Controlled Trials. Supplementary Table S8. Summary of reported outcomes of the included studies. Supplementary Table S9. Summary of findings table (single intervention compared with control for uptake cervical cancer screening). Supplementary Table S10. Summary of findings table (single in [file 12905_2023_2265_MOESM1_ESM.docx]

**Supplementary information**

**Title: Interventions to increase the uptake of cervical cancer screening in low- and middle-income countries: a systematic review and meta-analysis**

**Authors:**

Khaing Nwe Tin^1,2^, Chetta Ngamjarus^2*^, Siwanon Rattanakanokchai^2^, Jen Sothornwit^3^, Apiwat Aue-aungkul^3^, Aye Kyawt Paing^4^, Porjai Pattanittum^2^, Nampet Jampathong^5^, Pisake Lumbiganon^3^

**Affiliations:**

^1^Maternal and Reproductive Health Division, Department of Public Health, Ministry of Health, Naypyitaw, Myanmar

^2^ Department of Epidemiology and Biostatistics, Faculty of Public Health, Khon Kaen University, Khon Kaen, Thailand

^3^Department of Obstetrics and Gynecology, Faculty of Medicine, Khon Kaen University, Khon Kaen,Thailand

^4^Independent Researcher, Yangon, Myanmar

^5^Cochrane Thailand, Khon Kaen University, Khon Kaen, Thailand

***Corresponding author:**

Name: Dr. Chetta Ngamjarus

Address: Associate Professor, Department of Epidemiology and Biostatistics, Faculty of Public Health, Khon Kaen University, Thailand

E-Mail: nchett@kku.ac.th

**Supplementary information**

**Supplementary 1:** Differences between protocol and review**.**

**Supplementary 2:** Design effect calculation for cluster RCTs.

**Supplementary 3:** Other outcomes those are not included in comparisons.

**Supplementary Figure S1:** Effect of single intervention on uptake of cervical cancer screening when compared with control according to (A) type of intervention, (B) study setting, (C) region, and (D) publication year.

**Supplementary Figure S2:** Funnel plot of the effect of single intervention on uptake of cervical cancer screening when compared with control.

**Supplementary Figure S3:** Effect of single intervention on willingness to get cervical cancer screening when compared with control according to region.

**Supplementary Figure S4:** Effect of single intervention on knowledge score when compared with control according to (A) study setting and (B) region

**Supplementary Figure S5:** Effect of combined interventions on uptake of cervical cancer screening when compared with single intervention according to (A) type of intervention and (B) region

**Supplementary Figure S6:** Sensitivity analysis for effect of single intervention on willingness to get cervical cancer when compared with control by removing study with more than two domains of high risk of bias

**Supplementary Table S1:** Search strategy

**Supplementary Table S2:** List of included studies

**Supplementary Table S3:** List of excluded studies

**Supplementary Table S4:** List of studies ongoing and awaiting classification

**Supplementary Table S5:** PRISMA checklist

**Supplementary Table S6:** Included studies by types of interventions

**Supplementary Table S7:** Judgement for risk of bias assessment using the Cochrane Risk of Bias Tool for Randomized Controlled Trials

**Supplementary Table S8:** Summary of reported outcomes of the included studies

**Supplementary Table S9**: Summary of findings table (single intervention compared with control for uptake cervical cancer screening)

**Supplementary Table S10:** Summary of findings table (single intervention compared with other intervention for uptake cervical cancer screening)

**Supplementary Table S11:** Summary of findings table (combined interventions compared with single intervention for uptake cervical cancer screening)

**Supplementary 1: Differences between protocol and review**

At the protocol stage, we had planned to include all women in type of participants; however, we excluded those women who have diagnosed positive screening test and cervical cancer because we identified the effects of interventions to increase the uptake of screening services and excluded the follow up studies among screening test positive women.

We had planned to perform meta-analysis using fixed-effect model, if there was no substantial or considerable heterogeneity (I² 0% to 50%) and to perform subgroup analyses by using random-effect models and if substantial heterogeneity (I² 50% to 75%). In addition, we planned not to undertake meta-analysis if there was considerable heterogeneity (I² higher than 75%) or there was considerable variation in clinical or methodological among included studies; instead, we would provide brief narrative findings. However, we performed meta-analysis using random-effect model because the intervention effects appear to vary across studies. We did not combine treatment effects between included studies if it is not appropriate. When subgroup analyses could not identify the reasons of heterogeneities, we used the findings of analysis with random-effect models.

We had planned to perform subgroup analysis according to the different populations (women or health personnel or community), different type of interventions, duration of interventions, continents of origin and year of publication. However, there was only targeted to the women in all included trials and durations were irrelevant to make the subgroups, thus subgroup analyses were conducted by type of interventions, study settings, continents of origin and year of publication.

**Supplementary 2: Design effect calculation for cluster RCTs**

According to the Cochrane handbook <https://training.cochrane.org/handbook/current/chapter-23#section-23-1-4>, the design effect was calculated with the formula as below;
$1+\left( M-1 \right)\times ICC$ where *M* is the average cluster size and ICC is the intra-cluster correlation coefficient. M is calculated total no of participants (intervention + control) divided by total number of clusters (intervention + control).

**With ICC= 0.0295 (Hade 2010)** (Hade *et al.*, 2010)

**Romli 2020**

3 clusters in each arm; 101 participants in each arm

M = (101+101)$\div$(3+3)= 202$\div$6=33.667

1+ (M-1) ICC = 1 + (33.667-1) x 0.0295 =1.96

**Samami 2021**

1 cluster in each arm; 60 participants in each arm

M = (60+60)$\div$(1+1)= 120$\div$2=60


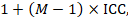


1+ (M-1) ICC = 1 + (60-1) x 0.02 = 1+ 59 x 0.0295 = 2.74

**Abu 2020**

4 clusters in each arm; 1062 vs 1078 participants

M= (1062+1078)$\div$(4+4)=267.5

1+ (M-1) ICC = 1 + (267.5-1) x 0.0295 = 8.86

**Abdullah 2013**

20 schools in each arm; 199 participants in each arm

M= (199+199)$\div$(20+20)=9.95

1+ (M-1) ICC = 1 + (9.95-1) x 0.0295 = 1.26

**Gizaw 2019**

11 clusters in each arm; 1213 vs 1143 participants

M= (1213+1143)$\div$(11+11)=107.09

1+ (M-1) ICC = 1 + (107.09-1) x 0.0295 = 4.13

**Huchko 2018**

6 clusters in each arm; 2943 vs 3538 participants

M= (2943+3538)$\div$(6+6)=540.08

1+ (M-1) ICC = 1 + (540.08-1) x 0.0295 = 16.90

**Supplementary 3: Other outcomes those are not included in comparisons**

**Outcome: Willingness to get cervical cancer screening (continuous)**

While two studies mentioned this outcome, one study with three arms (Kurt and Akyuz, 2019) was included in comparison 2 and 3. Another study (Koc, Ozdez and Topatan, 2019) measured with median score for the effects of education using the PRECEDE-PROCEED education model. The health motivation score of Healthy Lifestyle Scale 2 for Cervical Cancer and Pap Smear Test were found to be higher among women in the study group as compared with those in the control group (11 and 9; P=.001; 78 participants in each arm).

**Outcome: Attitude score**

Among two studies that mentioned the attitude score, one (Abadi, Vakilian and Safari, 2018) had very low mean and SD and lower score in intervention group than control group even though it was mentioned as the higher score was the more positive attitude (1.85± 61 vs 2.86±51.4; P=0.001); thus we could not include in meta-analysis. We also contacted to authors, however, did not received any reply. And, another one (Romli *et al.*, 2020)was identified as the lower score was the better attitude. It was not found the difference in attitude score between those who received the standardized educational program intervention and those in control group (MD -2.40, 95% CI -5.30 to 0.50; 206 women).

**Outcome: Satisfactory knowledge group**

Only one study (Sossauer *et al.*, 2014), with four domains of high risk of bias, mentioned the knowledge in group as women in the educational intervention group showed higher knowledge about HPV (124 out of 152, 81.6%) than women in the control group (15 out of 149, 10.1%) with p value<0.05; (RR 8.10, 95% CI 4.99 to 13.17; 301 women).

**Outcome: Cost effectiveness**

Among the three studies that mentioned the cost effectiveness outcome, one study (Rashid *et al.*, 2014) reported CEA (Cost Effective Analysis) for four arms; reminding with phone call, registered letter, SMS and routine postal letter to repeat the Pap test, and ICER (Incremental Cost Effective Ratio). In CEA, mean cost for repeating Pap test of all recall methods; phone call (Malaysian ringgit (RM) 95.60 [SD RM 0.15]), registered letter (RM 153.23 [SD RM 0.13]), SMS (RM 133.45 [SD RM 0.14]), reduced than routine postal letter (RM 156.23 [SD RM 0.11]). Based on ICEA, if the current recall method by sending letter will change to using phone call, there will be an additional cost saving of RM 22.53 to get a woman does a repeat pap smear; which highlights phone call was the most cost effective intervention.

Second one (Mezei *et al.*, 2018)presented the results of cost-effectiveness analysis for the intervention arm only. It was mentioned that HPV-ST (HPV screen and treat) method was the least costly and most effective strategy at all screening frequencies, dominating HPV-VIA (HPV followed by VIA) and VIA alone. The Incremental Cost Effectiveness Ratios (ICERs) of HPV-ST once, three times and five times per lifetime were US$130 per Years of Life Saved (YLS), US$240 per YLS and US$470 per YLS, respectively.

In addition, third study (Shen *et al.*, 2018) mentioned mean without SD. It shows that mean costs per woman screened at the Community Health Campaigns were slightly lower than mean costs per woman screened at the health clinics ($25.00 compared to $29.56); thus, cervical cancer screening at community health campaigns increased screening coverage and lowered per-women screening costs.

**Supplementary Figure S1:** Effect of single intervention on uptake of cervical cancer screening when compared with control according to (A) type of intervention, (B) study setting, (C) region, and (D) publication year

| **(A) Subgroup analysis by** **type of intervention**   | **(B) Subgroup analysis by** **study setting**   |
| --- | --- |
| **(C) Subgroup analysis by** **region**   | **(D) Subgroup analysis by** **publication year**   |

C stands for Cluster RCTs

The sample sizes of cluster RCTs are corrected with design effect estimated from ICC=0.029

**Supplementary Figure S2:** Funnel plot of the effect of single intervention on uptake of cervical cancer screening when compared with control

|  |
| --- |

**Supplementary Figure S3:** Effect of single intervention on willingness to get cervical cancer screening when compared with control according to region

| **Subgroup analysis by region**   |
| --- |

**Supplementary Figure S4:** Effect of single intervention on knowledge score when compared with control according to (A) study setting and (B) region

| **(A)** **Subgroup analysis by study setting**   |
| --- |
| **(B)** **Subgroup analysis by region**   |

C stands for Cluster RCTs

The sample sizes of cluster RCTs are corrected with design effect estimated from ICC=0.029

**Supplementary Figure S5:** Effect of combined interventions on uptake of cervical cancer screening when compared with single intervention according to (A) type of intervention and (B) region

| **(A)** **Subgroup analysis by type of intervention**   |
| --- |
| **(B)** **Subgroup analysis by region**   |

C stands for Cluster RCTs

The sample sizes of cluster RCTs are corrected with design effect estimated from ICC=0.029

**Supplementary Figure S6:** Sensitivity analysis for effect of single intervention on willingness to get cervical cancer when compared with control by removing study with more than two domains of high risk of bias

|  |
| --- |

**Supplementary Table S1: Search strategy**

**1. PubMed**

| #1 | health personnel[MH] |
| --- | --- |
| #2 | physician[MH] |
| #3 | nurse[MH] |
| #4 | medical technologist[MH] |
| #5 | “health care provider*”[TIAB] OR “healthcare provider*”[TIAB] OR “healthcare worker*” [TIAB] OR “health staff”[TIAB] OR “health professional*”[TIAB] OR “hospital staff*” [TIAB] OR “health worker*” [TIAB] OR “physician*” [TIAB] OR “nurse*” [TIAB] OR “nursing personnel*” [TIAB] OR “gynecologist*” [TIAB] OR “gynaecologist*” [TIAB] OR “medical laboratory personnel*” [TIAB] OR “medical technologist*” [TIAB] OR “clinical laboratory personnel*” [TIAB] OR “medical laboratory scientist*” [TIAB] OR “clinical laboratory scientist*” [TIAB] OR “medical laboratory technician*” [TIAB] OR “clinical laboratory technician*” [TIAB] OR “medical laboratory assistant*” [TIAB] |
| #6 | #1 OR #2 OR #3 OR #4 OR #5 |
| #7 | women[MH] |
| #8 | “women”[TIAB] OR “woman”[TIAB] |
| #9 | #7 OR #8 |
| #10 | “communit*”[TIAB] OR “village*”[TIAB] OR “local administration*”[TIAB] OR “famil*” [TIAB] OR ((“rural”[TIAB] OR “urban”[TIAB]) AND (“area”[TIAB] OR “setting”[TIAB] OR “settings”[TIAB])) |
| #11 | #6 OR #9 OR #10 |
| #12 | Uterine Cervical Neoplasms[MH] |
| #13 | “Cervical Neoplasm*” [TIAB] OR “Uterine Cervical Neoplasm”[TIAB] OR “Cervix Neoplasm*” [TIAB] OR “Cancer of the Cervix”[TIAB] OR “Cancer of the Uterine Cervix”[TIAB] OR “Cervical Cancer*”[TIAB] OR “Cancer of Cervix”[TIAB] |
| #14 | #12 OR #13 |
| #15 | Early Detection of Cancer[MH] |
| #16 | Mass Screening[MH] |
| #17 | “Screening”[TIAB] OR “Cancer Early Detection”[TIAB] OR “Early Diagnosis”[TIAB] |
| #18 | #15 OR #16 OR #17 |
| #19 | #14 AND #18 |
| #20 | “Vaginal Smear*”[TIAB] OR (“pap”[TIAB] AND (“smear”[TIAB] OR “test”[TIAB])) OR ((“cervical”[TIAB] OR “cervix”[TIAB]) AND “smear*”[TIAB]) OR “visual inspection with acetic acid”[TIAB] OR ((“human papilloma virus”[TIAB] OR “HPV DNA”[TIAB] OR “HPV”[TIAB]) AND “test*”[TIAB]) OR ((“cytology”[TIAB] or “cytobrush”[TIAB]) and “cervi*”[TIAB]) |
| #21 | #19 OR #20 |
| #22 | Controlled Clinical Trial[PT] |
| #23 | Randomized Controlled Trial[PT] |
| #24 | ((“randomized”[TIAB] OR “randomised”[TIAB]) AND (“controlled trial”[TIAB] OR “controlled trials”[TIAB])) OR (“RCT”[TIAB] OR “RCTs”[TIAB]) |
| #25 | “randomly”[TIAB] |
| #26 | #22 OR #23 OR #24 OR #25 |
| #27 | #11 AND #21 AND #26 |
| #28 | #11 AND #21 AND #26 AND 2000/01/01: 2021/9/30[dp] |

**2. CENTRAL**

| #1 | MeSH descriptor: [Health Personnel] explode all trees |
| --- | --- |
| #2 | MeSH descriptor: [Physicians] explode all trees |
| #3 | MeSH descriptor: [Nursing] explode all trees |
| #4 | MeSH descriptor: [Medical Laboratory Personnel] explode all trees |
| #5 | (health care provider* OR healthcare provider* OR healthcare worker*  OR “health staff” OR health professional* OR hospital staff* OR health worker* OR physician* OR nurse* OR nursing personnel* OR gynecologist* OR gynaecologist* OR medical laboratory personnel* OR medical technologist* OR clinical laboratory personnel* OR medical laboratory scientist* OR clinical laboratory scientist* OR medical laboratory technician* OR clinical laboratory technician* OR medical laboratory assistant*):ti,ab,kw |
| #6 | #1 OR #2 OR #3 OR #4 OR #5 |
| #7 | MeSH descriptor: [Women] explode all trees |
| #8 | “women” OR “woman” |
| #9 | #7 OR #8 |
| #10 | (communit* OR village* OR local administration* OR famil* OR ((“rural” OR “urban”) AND (“area” OR “setting” OR “settings”))):ti,ab,kw |
| #11 | #6 OR #9 OR #10 |
| #12 | MeSH descriptor: [Uterine Cervical Neoplasms] explode all trees |
| #13 | (Cervical Neoplasm* OR “Uterine Cervical Neoplasm” OR Cervix Neoplasm*  OR “Cancer of the Cervix” OR “Cancer of the Uterine Cervix” OR Cervical Cancer* OR “Cancer of Cervix”):ti,ab,kw |
| #14 | #12 OR #13 |
| #15 | MeSH descriptor: [Early Detection of Cancer] explode all trees |
| #16 | MeSH descriptor: [Mass Screening] explode all trees |
| #17 | (“Screening” OR “Cancer Early Detection” OR “Early Diagnosis”):ti,ab,kw |
| #18 | #15 OR #16 OR #17 |
| #19 | #14 AND #18 |
| #20 | (Vaginal Smear* OR (“pap” AND (“smear” OR “test” )) OR ((“cervical” OR “cervix”) AND smear*) OR “visual inspection with acetic acid” OR ((“human papilloma virus” OR “HPV DNA” OR “HPV”) AND test*) OR ((“cytology” or “cytobrush”) and cervi*)):ti,ab,kw |
| #21 | #19 OR #20 |
| #22 | #11 AND #21 |
| #24 | #11 AND #21 with Publication Year from 2000 to 2021, in Trials |

**3. ISI Web of Science**

| #1 | TS=(((health care provider*) OR (healthcare provider*) OR (healthcare worker*) OR (health staff) OR (health professional*) OR (hospital staff*) OR (health worker*) OR (physician*) OR (nurse*) OR (nursing personnel*) OR (gynecologist*) OR (gynaecologist*) OR (medical laboratory personnel*) OR (medical technologist*) OR (clinical laboratory personnel*) OR (medical laboratory scientist*) OR (clinical laboratory scientist*) OR (medical laboratory technician*) OR (clinical laboratory technician*) OR (medical laboratory assistant*) OR (health personnel))) |
| --- | --- |
| #2 | TS=((women) OR (woman)) |
| #3 | TS=((communit*) OR (village*) OR (local administration*) OR (famil*) OR (((rural) OR (urban)) AND ((area) OR (setting) OR (settings)))) |
| #4 | #1 OR #2 OR #3 |
| #5 | TS=((Cervical Neoplasm*) OR (Uterine Cervical Neoplasm*) OR (Cervix Neoplasm*) OR (Cancer of the Cervix) OR (Cancer of the Uterine Cervix) OR (Cervical Cancer*) OR (Cancer of Cervix)) |
| #6 | TS=((Screening) OR (Early Detection) OR (Early Diagnosis)) |
| #7 | #5 AND #6 |
| #8 | TS=((Vaginal Smear*) OR ((pap) AND ((smear) OR (test))) OR (((cervical) OR (cervix)) AND (smear*)) OR (visual inspection with acetic acid) OR (((human papilloma virus) OR (HPV DNA) OR (HPV)) AND (test*)) OR (((cytology) or (cytobrush)) and (cervi*))) |
| #9 | #7 OR #8 |
| #10 | TS=(Controlled Clinical Trial*) |
| #11 | TS=(Randomized Controlled Trial*) |
| #12 | TS=((((randomized) OR (randomized)) AND ((controlled trial) OR (controlled trials))) OR ((RCT) OR (RCTs))) |
| #13 | TS=(randomly) |
| #14 | #10 OR #11 OR #12 OR #13 |
| #15 | #4 AND #9 AND #14 |
| #16 | #4 AND #9 AND #14 and 2019 or 2018 or 2017 or 2016 or 2015 or 2014 or 2013  or 2012 or 2011 or 2010 or 2009 or 2008 or 2007 or 2006 or 2005 or 2004 or 2003 or 2002 or 2001 (Exclude – Publication Years) |

**4. Scopus**

| #1 | TITLE-ABS-KEY((health care provider*) OR (healthcare provider*) OR (healthcare worker*) OR {health staff} OR (health professional*) OR (hospital staff*) OR (health worker*) OR physician* OR nurse* OR (nursing personnel*) OR gynecologist* OR gynaecologist* OR (medical laboratory personnel*) OR (medical technologist*) OR (clinical laboratory personnel*) OR (medical laboratory scientist*) OR (clinical laboratory scientist*) OR (medical laboratory technician*) OR (clinical laboratory technician*) OR (medical laboratory assistant*) OR {health personnel}) |
| --- | --- |
| #2 | TITLE-ABS-KEY({women} OR {woman}) |
| #3 | TITLE-ABS-KEY(communit* OR village* OR (local administration*) OR famil* OR (({rural} OR {urban}) W/3 ({area} OR {setting} OR {settings}))) |
| #4 | #1 OR #2 OR #3 |
| #5 | TITLE-ABS-KEY((Cervical Neoplasm*) OR (Uterine Cervical Neoplasm*) OR (Cervix Neoplasm*) OR “Cancer of the Cervix” OR “Cancer of the Uterine Cervix” OR (Cervical Cancer*) OR “Cancer of Cervix”) |
| #6 | TITLE-ABS-KEY({Screening} OR {Early Detection} OR {Early Diagnosis}) |
| #7 | #5 AND #6 |
| #8 | TITLE-ABS-KEY((Vaginal Smear*)) |
| #9 | TITLE-ABS-KEY(({pap} W/2 ({smear} OR {test}))) |
| #10 | TITLE-ABS-KEY((cervical OR cervix) PRE/2 smear) |
| #11 | TITLE-ABS-KEY({visual inspection} PRE/1 {acetic acid}) |
| #12 | TITLE-ABS-KEY((“human papilloma virus” OR “HPV”) PRE/2 test*) |
| #13 | TITLE-ABS-KEY((“cytology” or “cytobrush”) W/2 cervi*) |
| #14 | #8 OR #9 OR #10 OR #11 OR #12 OR #13 |
| #15 | #7 OR #14 |
| #16 | TITLE-ABS-KEY((“Controlled Clinical Trial*”)) |
| #17 | TITLE-ABS-KEY((“Randomized Controlled Trial*”)) |
| #18 | TITLE-ABS-KEY(((“randomized” OR “randomized”) PRE/2 (“controlled trial” OR “controlled trials”)) OR RCT*) |
| #19 | TITLE-ABS-KEY({randomly}) |
| #20 | #16 OR #17 OR #18 OR #19 |
| #21 | #4 AND #15 AND #20 |
| #22 | #4 AND #15 AND #20 AND ( LIMITTO ( PUBYEAR , 2021 ) OR LIMITTO ( PUBYEAR , 2020 ) OR LIMITTO ( PUBYEAR , 2019 ) OR LIMITTO ( PUBYEAR , 2018 ) OR LIMITTO ( PUBYEAR , 2017 ) OR LIMITTO ( PUBYEAR , 2016 ) OR LIMIT-TO ( PUBYEAR , 2015 ) OR LIMIT TO ( PUBYEAR , 2014 ) OR LIMITTO ( PUBYEAR , 2013 ) OR LIMIT-TO ( PUBYEAR , 2012 ) OR LIMIT TO ( PUBYEAR , 2011 ) OR LIMIT TO ( PUBYEAR , 2009 ) OR LIMIT-TO ( PUBYEAR , 2008 ) OR LIMIT-TO ( PUBYEAR , 2007 ) OR LIMIT-TO ( PUBYEAR , 2006 ) OR LIMIT-TO ( PUBYEAR , 2005 ) OR LIMIT-TO ( PUBYEAR , 2004 ) OR LIMIT-TO ( PUBYEAR , 2003 ) OR LIMIT-TO ( PUBYEAR , 2002 ) OR LIMIT-TO ( PUBYEAR , 2001 ) OR LIMIT-TO ( PUBYEAR , 2000 ) ) |

**5. Ovid (Medline)**

| 1 | exp Health Personnel/ |
| --- | --- |
| 2 | exp Physicians/ |
| 3 | exp Nurses/ |
| 4 | exp Medical Laboratory Personnel/  medical technologist[MH] |
| 5 | (health care provider$ or healthcare provider$ or healthcare worker$ or "health staff" or health professional$ or hospital staff$ or health worker$ or physician$ or nurse$ or nursing personnel$ or gynecologist$ or gynaecologist$ or medical laboratory personnel$ or medical technologist$ or clinical laboratory personnel$ or medical laboratory scientist$ or clinical laboratory scientist$ or medical laboratory technician$ or clinical laboratory technician$ or medical laboratory assistant$).ab,kw,ti. |
| 6 | 1 or 2 or 3 or 4 or 5 |
| 7 | exp Women/ |
| 8 | ("women" or "woman").ab,kw,ti. |
| 9 | 7 or 8 |
| 10 | (communit$ or village$ or local administration$ or famil$ or (("rural" or "urban") and ("area" or "setting" or "settings"))).ab,kw,ti. |
| 11 | 6 or 9 or 10 |
| 12 | exp Uterine Cervical Neoplasms/ |
| 13 | (Cervical Neoplasm$ or "Uterine Cervical Neoplasm" or Cervix Neoplasm$ or "Cancer of the Cervix" or "Cancer of the Uterine Cervix" or Cervical Cancer$ or "Cancer of Cervix").ab,kw,ti. |
| 14 | 12 or 13 |
| 15 | exp "Early Detection of Cancer"/ |
| 16 | exp Mass Screening/ |
| 17 | ("Screening" or "Cancer Early Detection" or "Early Diagnosis").ab,kw,ti. - |
| 18 | 15 or 16 or 17 |
| 19 | 14 and 18 |
| 20 | (Vaginal Smear$ or ("pap" and ("smear" or "test")) or (("cervical" or "cervix") and smear$) or "visual inspection with acetic acid" or (("human papilloma virus" or "HPV DNA" or "HPV") and test$) or (("cytology" or "cytobrush") and cervi$)).ab,kw,ti. |
| 21 | 19 or 20 |
| 22 | Controlled Clinical Trial.pt. |
| 23 | Randomized Controlled Trial.pt. |
| 24 | (("randomized" or "randomised") and ("controlled trial" or "controlled trials")).mp. or ("RCT" or "RCTs").ab,kw,ti. [mp=title, abstract, original title, name of substance word, subject heading word, floating sub-heading word, keyword heading word, organism supplementary concept word, protocol supplementary concept word, rare disease supplementary concept word, unique identifier, synonyms] |
| 25 | "randomly".ab,kw,ti. |
| 26 | 22 or 23 or 24 or 25 |
| 27 | 11 and 21 and 26 |
| 28 | 27 |

**6. CINAHL**

| S1 | TI ((health care provider*) OR ( healthcare provider*) OR (healthcare worker*) OR “health staff” OR (health professional*) OR (hospital staff*) OR (health worker*) OR physician* OR nurse* OR (nursing personnel*) OR gynecologist* OR gynaecologist* OR (medical laboratory personnel*) OR (medical technologist*) OR (clinical laboratory personnel*) OR (medical laboratory scientist*) OR (clinical laboratory scientist*) OR (medical laboratory technician*) OR (clinical laboratory technician*) OR (medical laboratory assistant*) OR “health personnel”) OR AB ((health care provider*) OR ( healthcare provider*) OR (healthcare worker*) OR “health staff” OR (health professional*) OR (hospital staff*) OR (health worker*) OR physician* OR nurse* OR (nursing personnel*) OR gynecologist* OR gynaecologist* OR (medical laboratory personnel*) OR (medical technologist*) OR (clinical laboratory personnel*) OR (medical laboratory scientist*) OR (clinical laboratory scientist*) OR (medical laboratory technician*) OR (clinical laboratory technician*) OR (medical laboratory assistant*) OR “health personnel”) |
| --- | --- |
| S2 | TI (“women” OR “woman”) OR AB (“women” OR “woman”) |
| S3 | TI (communit* OR village* OR local administration* OR famil* OR ((“rural” OR “urban”) AND (“area” OR “setting” OR “settings”))) OR AB (communit* OR village* OR local administration* OR famil* OR ((“rural” OR “urban”) AND (“area” OR “setting” OR “settings”))) |
| S4 | S1 OR S2 OR S3 |
| S5 | TI ((Cervical Neoplasm*) OR (Uterine Cervical Neoplasm*) OR (Cervix Neoplasm*) OR “Cancer of the Cervix” OR “Cancer of the Uterine Cervix” OR (Cervical Cancer*) OR “Cancer of Cervix”) OR AB ((Cervical Neoplasm*) OR (Uterine Cervical Neoplasm*) OR (Cervix Neoplasm*) OR “Cancer of the Cervix” OR “Cancer of the Uterine Cervix” OR (Cervical Cancer*) OR “Cancer of Cervix”) |
| S6 | TI (“Screening” OR “Early Detection” OR “Early Diagnosis”) OR AB (“Screening” OR “Early Detection” OR “Early Diagnosis”) |
| S7 | S5 AND S6 |
| S8 | TI ((Vaginal Smear*) OR (“pap” AND (“smear” OR “test”)) OR ((“cervical” OR “cervix”) AND smear*) OR “visual inspection with acetic acid” OR ((“human papilloma virus” OR “HPV DNA” OR “HPV”) AND test*) OR ((“cytology” or “cytobrush”) and cervi*)) OR AB ((Vaginal Smear*) OR (“pap” AND (“smear” OR “test”)) OR ((“cervical” OR “cervix”) AND smear*) OR “visual inspection with acetic acid” OR ((“human papilloma virus” OR “HPV DNA” OR “HPV”) AND test*) OR ((“cytology” or “cytobrush”) and cervi*)) |
| S9 | S7 OR S8 |
| S10 | TI ((Controlled Clinical Trial*)) OR AB ((Controlled Clinical Trial*)) |
| S11 | TI ((Randomized Controlled Trial*)) OR AB ((Randomized Controlled Trial*)) |
| S12 | TI (((“randomized” OR “randomised”) AND (“controlled trial” OR “controlled trials”)) OR (“RCT” OR “RCTs”)) OR AB (((“randomized” OR “randomised”) AND (“controlled trial” OR “controlled trials”)) OR (“RCT” OR “RCTs”)) |
| S13 | TI “randomly” OR AB “randomly” |
| S14 | S10 OR S11 OR S12 OR S13 |
| S15 | S4 AND S9 AND S14 |
| S16 | S4 AND S9 AND S14 Published Date: 20000101-20211231 |

**7. Open Grey:** [**http://www.opengrey.eu/**](http://www.opengrey.eu/)

((health care provider$ OR healthcare provider$ OR healthcare worker$ OR "health staff" OR health professional$ OR hospital staff$ OR health worker$ OR physician$ OR nurse$ OR nursing personnel$ OR gynecologist$ OR gynaecologist$ OR medical laboratory personnel$ OR medical technologist$ OR clinical laboratory personnel$ OR medical laboratory scientist$ OR clinical laboratory scientist$ OR medical laboratory technician$ OR clinical laboratory technician$ OR medical laboratory assistant$) OR ("women" OR "woman") OR (communit$ OR village$ OR local administration$ OR famil$ OR (("rural" OR "urban") AND ("area" OR "setting" OR "settings")))) AND (((Cervical Neoplasm$ OR "Uterine Cervical Neoplasm" OR Cervix Neoplasm$ OR "Cancer of the Cervix" OR "Cancer of the Uterine Cervix" OR Cervical Cancer$ OR "Cancer of Cervix") AND ("Screening" OR "Cancer Early Detection" OR "Early Diagnosis")) OR (Vaginal Smear$ OR ("pap" AND ("smear" OR "test")) OR (("cervical" OR "cervix") AND smear$) OR "visual inspection with acetic acid" OR (("human papilloma virus" OR "HPV DNA" OR "HPV") AND test$) OR (("cytology" or "cytobrush") and cervi$))) AND ((("randomized" OR "randomised") AND ("controlled trial" OR "controlled trials")) OR ("RCT" OR "RCTs") OR "randomly")

**8. CNKI:** [**https://oversea.cnki.net**](https://oversea.cnki.net/)

AB=(('health care provider' + 'healthcare provider' + 'health professional' + 'physician' + 'nurse' + 'gynecologist' + 'gynaecologist' + 'medical laboratory' + 'health personnel') * ('Cervical Neoplasm' + 'Uterine Cervical Neoplasm' + 'Cervix Neoplasm' + 'Cervical Cancer' + 'Cancer of Cervix') * ('screening' + 'early detection' + 'early diagnosis' + 'pap smear' + 'pap test' + 'hpv test') * ('Controlled Clinical' + 'Randomized Controlled' + 'Randomised Controlled' + 'RCT'))

**Supplementary Table S2:** **Listof included studies**

| **Abdai 2018** |
| --- |
| 1. * Abadi MMM, Vakilian K, Safari V. Motivational interview on having Pap test among middle-aged women – A counseling service in primary care. Fam Med Prim Care Rev. 2018;20(2):101–5. |
| 1. Irct2016080729224N. The effect of motivational interviewing on testing for cervical cancer in middle-aged women. <http://www.who.int/trialsearch/Trial2.aspx?TrialID=IRCT2016080729224N1> [Internet]. 2016; Available from:  <https://www.cochranelibrary.com/central/doi/10.1002/central/CN-01797430/full> |
| **Abdullah 2013** |
| 1. Abdullah F, Rorke MO, Murray L, Su TT. Evaluation of a Worksite Cervical Screening Initiative to Increase Pap Smear Uptake in Malaysia : A Cluster Randomized Controlled Trial. Biomed Res Int. 2013;2013. |
| **Abu 2020** |
| 1. Abu SH, Woldehanna BT, Nida ET, Tilahun W, Gebremariam MY, Sisay MM. The role of health education on cervical cancer screening uptake at selected health centers in Addis Ababa. PLoS One [Internet]. 2020;215:1–10. Available from: <http://dx.doi.org/10.1371/journal.pone.0239580> |
| **Adonis 2016** |
| 1. Adonis L, Paramanund J, Basu D, Luiz J. Framing preventive care messaging and cervical cancer screening in a health-insured population in South Africa: Implications for population-based communication ? J Health Psychol. 2016; 1-11 |
| **Altinel 2019** |
| 1. * Altinel B, Akin B. The effect of multiple interventions for women at risk for cervical cancer on their health responsibility, beliefs regarding cervical cancer, and having screening: A randomized controlled experiment. Ann Oncol. 2019 Oct 1;30:v427. <https://www.sciencedirect.com/science/article/pii/-S0923753419592602?via%3Dihub> |
| 1. Atinel B, Akin B. The Effect of Multiple Interventions for Women at Risk for Cervical Cancer on Their Randomized Controlled Study Protocol. arastirma Res. 2020;17:70-7. |
| **Dinshaw 2008** |
| 1. Dinshaw K, Mishra G, Shastri S, Badwe R, Kerkar R, Ramani S, et al. Reducing the Worldwide Burden of Cancer Determinants of Compliance in a Cluster Randomised Controlled Trial on Screening of Breast and Cervix Cancer in Mumbai , India. Oncol 2007. 2008;73:154–61. |
| **Erwin 2019** |
| 1. * Erwin E, Aronson KJ, Day A, Ginsburg O, Macheku G, Feksi A, et al. SMS behaviour change communication and eVoucher interventions to increase uptake of cervical cancer screening in the Kilimanjaro and Arusha regions of Tanzania : a randomised , double-blind , controlled trial of effectiveness. BMJ Innov. 2019;5:28–34. |
| 1. NCT02680613 2016. Pilot Study of a Mobile Health Approach to Reduce Barriers to Cervical Cancer Screening in Tanzania. https://clinicaltrials.gov/show/NCT02680613 [Internet]. 2016; Available from: <https://www.cochranelibrary.com/central/doi/10.1002/central/CN-01555759/full> |
| **Gizaw 2019** |
| 1. Gizaw M, Teka B, Ruddies F, Abebe T, Kaufmann AM, Worku A, et al. Uptake of cervical cancer screening in Ethiopia by self-sampling HPV DNA compared to visual inspection with acetic acid: a cluster randomized trial. Am Assoc Cancer Res. 2019; |
| 1. Gizaw M, Ruddies F, Addissie A, Worku A, Abebe T, Teka B, et al. Abstract B103: Community-based uptake of self-sampling for HPV DNA-based testing for cervical cancer screening in Ethiopia: Preliminary findings of a cluster randomized trial. Cancer Epidemiol Prev Biomarkers [Internet]. 2020 Jun 1 [cited 2022 Feb 9];29(6 Supplement 1):B103–B103. Available from: <https://cebp.aacrjournals.org/content/29/6_Supplement_1/B103> |
| **Huchko 2018** |
| 1. * Huchko MJ, Ibrahim S, Blat C, Cohen CR. Cervical cancer screening through human papillomavirus testing in community health campaigns versus health facilities in rural western Kenya. 2018; |
| 1. Huchko MJ, Kahn JG, Smith JS, Hiatt RA, Cohen CR, Bukusi E. Study protocol for a cluster-randomized trial to compare human papillomavirus based cervical cancer screening in community-health campaigns versus health facilities in western Kenya. BMC Cancer [Internet]. 2017; 17 (1) (no pagination CC-Gynaecological, Neuro-oncology and Orphan Cancer).Available from: <https://www.cochranelibrary.com/central/doi/10.1002/central/CN-01442021/full> |
| 1. Huchko MJ OECY, Kahn JG, Huchko MJ, Olwanda E, Choi Y, Kahn JG. HPV-based cervical cancer screening in low-resource settings: maximizing the efficiency of community-based strategies in rural Kenya. Int J Gynecol Obstet [Internet]. 2020;148(3):386‐391. |
| 1. Olwanda E, Shen J. Comparison of patient flow and provider efficiency of two delivery strategies for HPV-based cervical cancer screening in Western Kenya : a time and motion study. Glob Health Action [Internet]. 2018;11(1). Available from: <https://doi.org/10.1080/16549716.2018.1451455> |
| 1. Shen J, Olwanda E, Kahn JG, Huchko MJ. Cost of HPV screening at community health campaigns ( CHCs ) and health clinics in rural Kenya. BMC Health Serv Res. 2018;18(378):1–10. |
| **Koc 2019** |
| 1. Koc Z, Ozdez EK, Topatan S. The Impact of Education About Cervical Cancer and Human Papillomavirus on Women’s Healthy Lifestyle Behaviors and Beliefs. Wolters Kluwer Heal Inc. 2019;42(2). |
| **Kurt 2019** |
| 1. Kurt G, Akyuz A. Evaluating the Effectiveness of Interventions on Increasing Participation in Cervical Cancer Screening. J Nurs Res. 2019;27(5):1–11. |
| **Mitchell 2015** |
| 1. Mitchell S, Moses H, Seikubo M, Mwesigwa D, Singer J. Examining HPV prevalence and uptake of cervical cancer screening among HIV positive and negative women partcipating in a pilot RCT in Uganda comparing self-collection based HPV testing to VIA. Sex Transm Infect. 2015;18(Suppl 2):1–2. |
| **Mittra 2010** |
| 1. Mittra I, Mishra GA, Singh S, Aranke S, Notani P, Badwe R, et al. Cancer screening in Mumbai , India : methodology and interim results after three rounds of screening. Int J cancer.2010;984:976–84. |
| **Modibbo 2017** |
| 1. Modibbo F, Iregbu KC, Okuma J, Leeman A, Kasius A, Koning M De, et al. Randomized trial evaluating self-sampling for HPV DNA based tests for cervical cancer screening in Nigeria. Infect Agent Cancer. 2017;12(11):1–9. |
| **Moses 2015** |
| 1. Moses E, Pedersen H, Mitchell S, Sekikubo M. Towards the best model for cervical cancer screening in low and middle inclome countries: Results from a pilot randomized controlled trial comparing self-collected HPV testing with VIA in Uganda. Int J Gynecol Obstet 131,. 2015;Suppl 5:72–313. |
| 1. * Moses E, Pedersen HN, Mitchell SM, Sekikubo M, Mwesigwa D, Singer J, et al. Uptake of community-based , self-collected HPV testing vs . visual inspection with acetic acid for cervical cancer screening in Kampala , Uganda : preliminary results of a randomised controlled trial. Trop Med Int Heal. 2015;20(10):1355–67. |
| 1. Mezei AK, Pedersen HN, Sy S, Regan C, Mitchell-foster SM, Byamugisha J, et al. Community-based HPV self-collection versus visual inspection with acetic acid in Uganda : a cost-effectiveness analysis of the ASPIRE trial. BMJ Open. 2018;1–12. |
| **Okeke 2016** |
| 1. Okeke EN. Does Price Affect the Demand for Information about New Health Technologies ? Evidence from a Field Experiment in Nigeria. Univ Chicago Press.2016; |
| **Okunade 2021** |
| 1. * Okunade KS, Soibi-harry A, John-olabode S, Adejimi AA, Allsop MJ. Impact of Mobile Technologies on Cervical Cancer Screening Practices in Lagos , Nigeria ( mHealth-Cervix ): A Randomized Controlled Trial abstract. JCO Glob Oncol. 2021; |
| 1. Okunade KS, Salako O, Adejimi AA, Akinsola OJ, Fatiregun O, Adenekan MA, et al. Impact of mobile technologies on cervical cancer screening practices in Lagos , Nigeria ( mHealth-Cervix ): Protocol for a randomised controlled trial [ version 1 ; peer review : 2 approved ]. F1000Research. 2020;1–9. |
| **Rashid 2014** |
| 1. * Rashid RMA, Ramli S, John J, Dahlui M. Cost Effective Analysis of Recall Methods for Cervical Cancer Screening in Selangor - Results from a Prospective Randomized Controlled Trial. Asian Pac J Cancer Prev. 2014;15(13):5143–7. |
| 1. Rashid RMA, Mohamed M, Hamid ZA, Dahlui M. Is the Phone Call the Most Effective Method for Recall in Cervical Cancer Screening ? - Results from a Randomised Control Trial. Asian Pac J Cancer Prev. 2014;15(13):5143–7. |
| 1. Abdul Rashid RM, Dahlui M. Study protocol for the most effective recall method in a cervical cancer screening program in Klang, Malaysia. Asian Pacific J cancer Prev[Internet].2013;14(10):5867–70.Available from: <https://www.cochranelibrary.com/central/doi/10.1002/central/CN-01047097/full> |
| 1. IRCT2013061113632N1 2013. Call-Recall System for Pap Smear screening in Klang: patients’ Response and Cost Effective Analysis of Different Types of Reminder on the Patients’ Response Rates. http://www.who.int/trialsearch/ Trial2.aspx?TrialID=IRCT2013061113632N1 [Internet]. 2013; Available from: [https://www.cochranelibrary.com/central/ doi/10.1002/central/CN-01865686/full](https://www.cochranelibrary.com/central/%20doi/10.1002/central/CN-01865686/full) |
| **Risi 2004** |
| 1. Risi L, Bindman JP, Campbell OMR, Imrie J, Everett K, Bradley J, et al. Media interventions to increase cervical screening uptake in South Africa : an evaluation study of effectiveness. Health Educ Res. 2004;19(4):457–68. |
| **Romli 2020** |
| 1. Romli R, Shahabudin S, Saddki N, Mokhtar N, Romli R SSSN, Mokhtar N, et al. Effectiveness of a health education program to improve knowledge and attitude towards cervical cancer and pap smear: A controlled community trial in Malaysia. Asian Pacific J Cancer Prev [Internet]. 2020;21(3):853‐859. Available from: <https://pubmed.ncbi.nlm.nih.gov/32212817/> |
| **Rosser 2015** |
| 1. Rosser JI, Njoroge B, Huchko MJ. Patient Education and Counseling Changing knowledge , attitudes , and behaviors regarding cervical cancer screening : The effects of an educational intervention in rural Kenya. Patient Educ Couns [Internet]. 2015;6–11. Available from: <http://dx.doi.org/10.1016/j.pec.2015.03.017> |
| **Samami 2021** |
| 1. Samami E, Seyedi-Andi SJ, Bayat B, Shojaeizadeh D, Tori NA. The effect of educational intervention based on the health belief model on knowledge, attitude, and function of women about Pap smear test at Iranian health centers: A randomized controlled clinical trial. J Educ Health Promot. 2021;10(1). |
| **Shokoohi 2020** |
| 1. Shokoohi M, Jamshidimanesh M, Ranjbar H, Saffari M, Motamed A. The effectiveness of a model-based health education program on protective behavior against human papillomavirus in female drug abusers: a randomized controlled trial. HIV AIDS Rev. 2020;19(1):16–23. |
| **Sossauer 2014** |
| 1. Sossauer G, Zbinden M, Tebeu P, Fosso K, Untiet S, Vassilakos P, et al. Impact of an Educational Intervention on Women ’ s Knowledge and Acceptability of Human Papillomavirus Self-Sampling : A Randomized Controlled Trial in. PLoS One. 2014;9(10):1–8. |

* The primary reference for the study

**Supplementary Table S3: List of excluded studies**

| **Studies excluded due to wrong setting** |
| --- |
| 1. ACTRN12613001003763. A Randomized control trial to evaluate Human papillomavirus (HPV) DNA self-sampling as a primary cervical screening test in women. http://www.who.int/trialsearch/Trial2.aspx?TrialID=ACTRN12613001003763 [Internet]. 2013; Available from: <https://www.cochranelibrary.com/central/doi/10.1002/central/CN-01819138/full> |
| 1. Arrossi S, Thouyaret L, Herrero R, Campanera A, Magdaleno A, Cuberli M, et al. Effect of self-collection of HPV DNA offered by community health workers at home visits on uptake of screening for cervical cancer (the EMA study): a population-based cluster-randomised trial. lancet Glob Heal [Internet]. 2015;3(2 CC-Gynaecological, Neuro-oncology and Orphan Cancer):e85–94. Available from: <https://www.cochranelibrary.com/central/doi/10.1002/central/CN-01049240/full> |
| 1. Batal H, Biggerstaff S, Dunn T, Mehler PS. Cervical cancer screening in the urgent care setting. J Gen Intern Med [Internet]. 2000;15(6 CC-Anaesthesia CC-SR-CANCER CC-Effective Practice and Organisation of Care CC-Gynaecological, Neuro-oncology and Orphan Cancer):389–94. Available from: <https://www.cochranelibrary.com/central/doi/10.1002/central/CN-00298129/full> |
| 1. Breitkopf CR, Dawson L, Grady JJ, Breitkopf DM, Nelson-Becker C, Snyder RR. Intervention to improve follow-up for abnormal Papanicolaou tests: a randomized clinical trial. Heal Psychol [Internet]. 2014;33(4):307–16. Available from: [https://www.cochranelibrary.com/central/doi/10.1002/ central/CN-00984447/full](https://www.cochranelibrary.com/central/doi/10.1002/%20central/CN-00984447/full) |
| 1. Burack RC, Gimotty PA, George J, McBride S, Moncrease A, Simon MS, et al. How reminders given to patients and physicians affected pap smear use in a health maintenance organization: results of a randomized controlled trial. Cancer [Internet]. 1998;82(12 CC-SR-CANCER CC-Consumers and Communication CC-Effective Practice and Organisation of Care CC-Gynaecological, Neuro-oncology and Orphan Cancer):2391–400. Available from: https://www.cochranelibrary.com/central/doi/10.1002/central/CN-00151868/full |
| 1. ChiCTR1800017227. A multimedia educational program to increase utilisation rate of cervical cancer screening among South Asian women.   http://www.who.int/trialsearch/Trial2.aspx?TrialID=ChiCTR1800017227 [Internet]. 2018; Available from: <https://www.cochranelibrary.com/central/doi/10.1002/central/CN-01909215/full> |
| 1. Decker KM, Turner D, Demers AA, Martens PJ, Lambert P, Chateau D. Evaluating the effectiveness of cervical cancer screening invitation letters. J Womens Health (Larchmt) [Internet]. 2013;22(8 CC-Gynaecological, Neuro-oncology and Orphan Cancer):687–93. Available from: https://www.cochranelibrary.com/central/doi/10.1002/central/CN-00962955/full |
| 1. Effectiveness of three interventions in improving adherence to cervical cancer screening. Eur J cancer Prev 25 (pp 423-429), 2016 Date Publ 2016 [Internet]. Available from: https://www.cochranelibrary.com/central/doi/10.1002/central/ CN-01197170/full |
| 1. Elder JP, Haughton J, Perez LG, Martinez ME, De la Torre CL, Slymen DJ, et al. Promoting cancer screening among churchgoing Latinas: fe en Accion/faith in action. Health Educ Res [Internet]. 2017;32(2 CC-Gynaecological, Neuro-oncology and Orphan Cancer):163–73. Available from: <https://www.cochranelibrary.com/central/doi/10.1002/central/CN-01655165/full> |
| 1. Elfstrom KM, Sundstrom K, Andersson S, Bzhalava Z, Carlsten Thor A, Gzoul Z, et al. Increasing participation in cervical screening by targeting long-term nonattenders: randomized health services study. Int J cancer [Internet]. 2019; |
| 1. Giorgi Rossi P, Fortunato C, Barbarino P, Boveri S, Caroli S, Del Mistro A, et al. Self-sampling to increase participation in cervical cancer screening: an RCT comparing home mailing, distribution in pharmacies, and recall letter. Br J Cancer [Internet]. 2015;112(4 CC-Gynaecological, Neuro-oncology and Orphan Cancer):667–75. Available from: https://www.cochranelibrary.com/central/ doi/10.1002/central/CN-01052141/full |
| 1. Greimel ER, Gappmayer-Löcker E, Girardi FL, Huber HP. Increasing women’s knowledge and satisfaction with cervical cancer screening. J Psychosom Obstet Gynaecol [Internet]. 1997;18(4 CC-HS-DEPRESSN CC-SR-CANCER CC-Consumers and Communication CC-Gynaecological, Neuro-oncology and Orphan Cancer CC-HS-HANDSRCH):273–9. Available from: <https://www.cochranelibrary.com/central/doi/10.1002/central/CN-00147075/full> |
| 1. Hwang AS, Harding AS, Chang Y, O’Keefe SM, Horn DM, Clark AL. An Audit and Feedback Intervention to Improve Internal Medicine Residents’ Performance on Ambulatory Quality Measures: a Randomized Controlled Trial. Popul Health Manag [Internet]. 2019; Available from: https://www.cochranelibrary.com/central/doi/10.1002/central/CN-01939117/full |
| 1. IRCT201304087132N. The effect of educational intervention based on a model of behavior change, integrated of Protection Motivation Theory and Implementation Intention for perform the Pap test in women. <http://www.who.int/trialsearch/Trial2.aspx>? |
| 1. ISRCTN52303479. Strategies to increase cervical screening uptake at first invitation. http://www.who.int/trialsearch/Trial2.aspx?TrialID=ISRCTN52303479 [Internet]. 2011; Available from: [https://www.cochranelibrary.com/central/ doi/10.1002/central/CN-01815923/full](https://www.cochranelibrary.com/central/%20doi/10.1002/central/CN-01815923/full) |
| 1. Jibaja-Weiss ML, Volk RJ, Kingery P, Smith QW, Holcomb JD. Tailored messages for breast and cervical cancer screening of low-income and minority women using medical records data. Patient Educ Couns [Internet]. 2003;50(2 CC-Breast Cancer CC-Effective Practice and Organisation of Care CC-Gynaecological, Neuro-oncology and Orphan Cancer):123–32. Available from: https://www.cochranelibrary.com/central/doi/10.1002/central/CN-00438002/full |
| 1. JPRN-UMIN000019529. The effects of message framing on cervical cancer screening: a randomized controlled trial. http://www.who.int/trialsearch/ Trial2.aspx?TrialID=JPRN-UMIN000019529 [Internet]. 2015; Available from: <https://www.cochranelibrary.com/central/doi/10.1002/central/CN-01846362/full> |
| 1. Margolis KL, Lurie N, McGovern PG, Tyrrell M, Slater JS. Increasing breast and cervical cancer screening in low-income women. J Gen Intern Med [Internet]. 1998;13(8 CC-Breast Cancer CC-SR-CANCER CC-Consumers and Communication CC-Gynaecological, Neuro-oncology and Orphan Cancer):515–21. Available from: https://www.cochranelibrary.com/central/ doi/10.1002/central/CN-00154682/full |
| 1. NCT02005510. Randomized Trial of In-Home Cervical Cancer Screening in Underscreened Women. https://clinicaltrials.gov/show/NCT02005510 [Internet]. 2013; Available from: <https://www.cochranelibrary.com/central/doi/10.1002/central/CN-01479582/full> |
| 1. NCT02427399. Conducting Outreach to Improve Cervical Cancer Screening Rates Among Underscreened Patients. https://clinicaltrials.gov/show/NCT02427399 [Internet]. 2015; Available from: <https://www.cochranelibrary.com/central/doi/10.1002/central/CN-02036801/full> |
| 1. Nicolau AIO, Lima TM, Vasconcelos CTM, Carvalho FHC, Aquino PS, Pinheiro AKB. Telephone interventions in adherence to receiving the Pap test report: a randomized clinical trial. Rev Lat Am Enfermagem [Internet]. 2017;25:e2948–e2948. Available from: https://www.cochranelibrary.com/central/doi/10.1002/central/CN-01621722/full |
| 1. Peitzmeier SM, Khullar K, Potter J. Effectiveness of four outreach modalities to patients overdue for cervical cancer screening in the primary care setting: a randomized trial. Cancer causes Control [Internet]. 2016;27(9 CC-Gynaecological, Neuro-oncology and Orphan Cancer):1081–91. Available from: https://www.cochranelibrary.com/central/doi/10.1002/central/CN-01382578/full |
| 1. Percac-Lima S, Ashburner JM, Zai AH, Chang Y, Oo SA, Guimaraes E, et al. Patient Navigation for Comprehensive Cancer Screening in High-Risk Patients Using a Population-Based Health Information Technology System: a Randomized Clinical Trial. JAMA Intern Med [Internet]. 2016;176(7 CC-Gynaecological, Neuro-oncology and Orphan Cancer):930–7. Available from: <https://www.cochranelibrary.com/central/doi/10.1002/central/CN-01177759/full> |
| 1. Pierce M, Lundy S, Palanisamy A, Winning S, King J. Prospective randomised controlled trial of methods of call and recall for cervical cytology screening. BMJ [Internet]. 1989;299(6692 CC-SR-CANCER CC-Consumers and Communication CC-Effective Practice and Organisation of Care CC-Gynaecological, Neuro-oncology and Orphan Cancer):160–2. Available from: https://www.cochranelibrary.com/central/doi/10.1002/central/CN-00061864/full |
| 1. Rivers SE, Salovey P, Pizarro DA, Pizarro J, Schneider TR. Message framing and pap test utilization among women attending a community health clinic. J Health Psychol [Internet]. 2005;10(1 CC-Gynaecological, Neuro-oncology and Orphan Cancer CC-SR-HEALTHP):65–77. Available from: https://www.cochranelibrary.com/central/doi/10.1002/central/CN-00560452/full |
| 1. Ruffin MT, Gorenflo DW, Iv RMT, Dw G, Ruffin Iv MT. Interventions fail to increase cancer screening rates in community-based primary care practices. Prev Med (Baltim) [Internet]. 2004;39(3):435–40. |
| 1. Torres-Mejía G, Salmerón-Castro J, Téllez-Rojo MM, Lazcano-Ponce EC, Juárez-Márquez SA, Torres-Torija I, et al. Call and recall for cervical cancer screening in a developing country: a randomised field trial. Int J cancer [Internet]. 2000;87(6 CC-SR-BEHAVMED CC-SR-CANCER CC-Consumers and Communication CC-Gynaecological, Neuro-oncology and Orphan Cancer):869–73. Available from: https://www.cochranelibrary.com/central/doi/10.1002/central/CN-00299150/full |
| 1. Valanis BG, Glasgow RE, Mullooly J, Vogt TM, Whitlock EP, Boles SM, et al. Screening HMO women overdue for both mammograms and pap tests. Prev Med (Baltim) [Internet]. 2002;34(1 CC-Breast Cancer CC-Consumers and Communication CC-Effective Practice and Organisation of Care CC-Gynaecological, Neuro-oncology and Orphan Cancer):40–50. Available from: https://www.cochranelibrary.com/central/doi/10.1002/central/CN-00376513/full |
| 1. Vogt TM, Glass A, Glasgow RE, La Chance PA, Lichtenstein E, Tm V, et al. The safety net: a cost-effective approach to improving breast and cervical cancer screening. J Women’s Heal [Internet]. 2003;12(8):789–98. Available from: https://www.cochranelibrary.com/central/doi/10.1002/central/CN-00458578/full |
| 1. Wikström I, Lindell M, Sanner K, Wilander E, Wikstrom I. Self-sampling and HPV testing or ordinary Pap-smear in women not regularly attending screening: a randomised study. Br J Cancer [Internet]. 2011;105(3 CC-Gynaecological, Neuro-oncology and Orphan Cancer):337–9. Available from: https://www.cochranelibrary.com/central/doi/10.1002/central/CN-00798903/full |
| 1. Wong CL, So WKW, Chan DNS, Choi KC, Rana T. A community health worker-led multimedia intervention to increase cervical cancer screening uptake among South Asian women: study protocol for a cluster randomized wait-list controlled trial. Cochrane Central Register of Controlled Trials (CENTRAL) [Internet]. 2019; 20(1). Available from: <https://www.cochranelibrary.com/central/doi/10.1002/central/CN-01943261/full> |
| 1. Wong EL, Chan PK, Chor JS, Cheung AW, Huang F, Wong SY. Evaluation of the Impact of Human Papillomavirus DNA Self-sampling on the Uptake of Cervical Cancer Screening. Cancer Nurs [Internet]. 2016;39(1 CC-Gynaecological, Neuro-oncology and Orphan Cancer):E1–11. Available from: https://www.cochranelibrary.com/central/doi/10.1002/central/CN-01128070/full |
| **Studies excluded due to wrong study design** |
| 1. Amimo F, Moon TD, Magit A, Sacarlal J. Impact of Per Capita Income on the Effectiveness of School-Based Health Education Programs to Promote Cervical Cancer Screening Uptake in Southern Mozambique. J Glob Infect Dis [Internet]. 2018;10(3):152+-152+. Availablefrom: https://www.ncbi.nlm.nih.gov/pmc/articles/PMC6100336/ |
| 1. Arrossi S. Introduction of HPV self-collection in Argentina, main results, and lessons. Cancer Epidemiol biomarkers Prev. 2020;29(6 SUPPL 2). |
| 1. Azlina FA SS, Budiati T. Female health education package enhances knowledge, attitudes, and self-efficacy of housewives in cervical cancer screening. Enferm Clin. 31 Suppl 2. |
| 1. Bebis H, Reis N, Yavan T, Bayrak D, Unal A, Bodur S. Effect of health education about cervical cancer and papanicolaou testing on the behavior, knowledge, and beliefs of Turkish women. Int J Gynecol cancer [Internet]. 2012;22(8):1407–12. Available from: https://www.cochranelibrary.com/central/doi/10.1002/central/ CN-00879110/full |
| 1. Burack RC, Gimotty PA, Simon M, Moncrease A, Dews P. The effect of adding Pap smear information to a mammography reminder system in an HMO: results of randomized controlled trial. Prev Med (Baltim) [Internet]. 2003;36(5 CC-Breast Cancer CC-Consumers and Communication CC-Effective Practice and Organisation of Care CC-Gynaecological, Neuro-oncology and Orphan Cancer):547–54. Available from: https://www.cochranelibrary.com/central/ doi/10.1002/central/CN-00436561/full |
| 1. Chalapati W, Chumworathayi B. Can a home-visit invitation increase Pap smear screening in Samliem, Khon Kaen, Thailand? Asian Pacific J cancer Prev [Internet]. 2007;8(1 CC-Gynaecological, Neuro-oncology and Orphan Cancer):119–23. Available from: https://www.cochranelibrary.com/central/ doi/10.1002/central/CN-00588567/full |
| 1. Chigbu CO, Onyebuchi AK, Onyeka TC, Odugu BU, Dim CC. The impact of community health educators on uptake of cervical and breast cancer prevention services in Nigeria. Int J Gynaecol Obstet [Internet]. 2017;137(3 CC-Gynaecological, Neuro-oncology and Orphan Cancer):319–24. Available from: https://www.cochranelibrary.com/central/doi/10.1002/central/CN-01456920/full |
| 1. Chumworathayi B, Yuenyao P, Luanratanakorn S, Pattamadilok J, Chalapati W, Na-Nhongkai C. Can an appointment-letter intervention increase pap smear screening in Samliem, Khon Kaen, Thailand? Asian Pacific J cancer Prev [Internet]. 2007;8(3 CC-Gynaecological, Neuro-oncology and Orphan Cancer):353–6. Available from: https://www.cochranelibrary.com/central/ doi/10.1002/central/CN-00637415/full |
| 1. Daryani S, Shojaeezadeh D, Batebi A, Charati JY, Naghibi A. The effect of education based on a health belief model in women’s practice with regard to the Pap smear test. J CANCER POLICY. 2016;8:51–6. |
| 1. Eghbal SB, Karimy M, Kasmaei P, Roshan ZA, Valipour R, Attari SM, et al. Evaluating the effect of an educational program on increasing cervical cancer screening behavior among rural women in Guilan, Iran. BMC Womens Health [Internet]. 2020 Jul 20;20(1):1–9. Available from: https://search.ebscohost.com/ login.aspx?direct=true&db=ccm&AN=144673891&site=ehost-live |
| 1. Han L, Chang X, Song P, Gao L, Zhang Y, An L, et al. An on-going study of three different cervical cancer screening strategies based on primary healthcare facilities in Beijing China. J Infect Public Health. 2020 Apr;13(4):577–83. |
| 1. IRCT2013123016006N. The effect of an educational intervention based on the Health Belief Model on the refer of rural women to Shaft selected Health Center for Pap Smear test. http://www.who.int/trialsearch/Trial2.aspx?TrialID= IRCT2013123016006N1 [Internet]. 2014; Available from: https://www.cochranelibrary.com/central/doi/10.1002/central/CN-01849243/full |
| 1. IRCT2014092918120N. Effect of an intervention based on I-Change model in comparison with existing health education methods on change of intention of women in utilizing preventive services of cancer (Cervical Cancer). http://www.who.int/trialsearch/Trial2.aspx?TrialID=IRCT2014092918120N1 [Internet]. 2014; Available from: https://www.cochranelibrary.com/central/ doi/10.1002/central/CN-01859270/full |
| 1. IRCT2015110410426N. The effect of group counseling based on Health Belief Model on cervical cancer screening among rural women in Kabudarahang city in 2015-2016. http://www.who.int/trialsearch/Trial2.aspx?TrialID= IRCT2015110410426N8 [Internet]. 2015; Available from: https://www.cochranelibrary.com/central/doi/10.1002/central/CN-01866654/full |
| 1. IRCT20160830029608N. The survey factors associated with Pap test screening and educational intervention based on the theory of planned behavior in women. http://www.who.int/trialsearch/Trial2.aspx?TrialID=IRCT20160830029608N3 [Internet]. 2019; Available from: https://www.cochranelibrary.com/central/ doi/10.1002/central/CN-01948896/full |
| 1. IRCT2017100729954N. The Effect of Motivational Interview on the Frequency of Cervical Cancer and Breast Cancer Screening. http://www.who.int/ trialsearch/Trial2.aspx? TrialID=IRCT2017100729954N4 [Internet]. 2017; Available from: https://www.cochranelibrary.com/central/doi/10.1002/central/ CN-01896604/full |
| 1. Manglardi EE, Lendore J, Balhotra K, Fields PJ, Bahadoor-Yetman A, McGill FM. Self Screening as an Alternative Method of Cervical Screening [5D]. Obstet Gynecol. 2019;133 Suppl. |
| 1. Manglardi EE, Lendore J, Balhotra K, Fields PJ. Self-Screening as an Alternative Method of Cervical Screening. American College of Obstetrics and Gynecologists, 133(5 (Supplement)) 2019; 42–3. |
| 1. NCT02124252. Community-Driven Cervical Cancer Prevention in Western Kenya. https://clinicaltrials.gov/show/NCT02124252 [Internet]. 2014; Available from: https://www.cochranelibrary.com/central/doi/10.1002/central/ CN-02044613/full |
| 1. Ndikom CM, Ofi BA, Omokhodion FO, Adedokun BO. Effects of educational intervention on women’s knowledge and uptake of cervical cancer screening in selected hospitals in Ibadan, Nigeria. Int J Heal Promot Educ [Internet]. 2017;55(5–6):259–71. Available from: https://www.tandfonline.com/doi/full/ 10.1080/14635240.2017.1372693 |
| 1. Sankaranarayanan R, Dinshaw K, Nene BM, Ramadas K, Esmy PO, Jayant K, et al. Cervical and oral cancer screening in India. J Med Screen. 2006;13(1): S35–8. |
| 1. Seay J COTDGMBAAAPLK-ST, Kobetz E, Seay J, Carrasquillo O, Trevil D, Gonzalez M, et al. Implementing Two Randomized Pragmatic Trials of HPV Self-sampling among Underserved Women: challenges and Lessons Learned. Prog Community Heal Partnerships Res Educ Action [Internet]. 2020;14(1):55–62. Available from: https://pubmed.ncbi.nlm.nih.gov/32280123/ |
| 1. Tang JH SJSMSGLMVCECJSENBBRMMCM, Chinula L, Tang JH, Smith JS, McGue S, Gadama L, et al. Prevention of cervical cancer through two HPV-based screen-and-treat implementation models in Malawi: protocol for a cluster randomized feasibility trial. Pilot Feasibility Stud [Internet]. 2021;7(1). |
| **Studies excluded due to wrong population** |
| 1. Andersson S, Belkic K, Mints M, Ostensson E. Acceptance of Self-Sampling Among Long-Term Cervical Screening Non-Attenders with HPV-Positive Results: Promising Opportunity for Specific Cancer Education. J Cancer Educ [Internet]. 2021;36(1):126–33. Available from: https://pubmed.ncbi.nlm.nih.gov/31522376/ |
| 1. Hellsten C EABGFO, Borgfeldt C, Hellsten C, Ernstson A, Bodelsson G, Forslund O, et al. Equal prevalence of severe cervical dysplasia by HPV self-sampling and by midwife-collected samples for primary HPV screening: a randomised controlled trial. Eur J cancer Prev. 2021 Jul;30(4):334–40. |
| 1. Linde DS, Andersen MS, Mwaiselage J, Manongi R, Kjaer SK, Rasch V, et al. Effectiveness of One-Way Text Messaging on Attendance to Follow-Up Cervical Cancer Screening Among Human Papillomavirus-Positive Tanzanian Women (Connected2Care): Parallel-Group Randomized Controlled Trial. J Med Internet Res [Internet]. 2020 Apr;22(4):N.PAG-N.PAG. Available from: https://search.ebscohost.com/login.aspx?direct=true&db=ccm&AN=142621809&site=ehost-live |
| 1. Linde DS, Andersen MS, Mwaiselage JD, Manongi R, Kjaer SK, Rasch V. Text messages to increase attendance to follow-up cervical cancer screening appointments among HPV-positive Tanzanian women (Connected2Care): study protocol for a randomised controlled trial. Cochrane Central Register of Controlled Trials (CENTRAL) [Internet]. 2017;18(1) (no pagination). Available from: https://www.cochranelibrary.com/central/doi/ 10.1002/central/CN01432446/full |
| 1. Moscicki A-B, Chang C, Vangala S, Zhou X, Elashoff DA, Dehlendorf C, et al. Effect of 2 interventions on cervical cancer screening guideline adherence. Am J Prev Med [Internet]. 2021;60(5):666–73. Available from: https://www.scopus.com/ inward/-record.uri?eid=2-s2.085101366630&doi=10.1016%2Fj.amepre.2020.11.015& partnerID-=40&md5=cda53f1f6708 e4e068222ae4aca574e6 |
| 1. NCT04794660. The Study for the “Cervical Cancer Screening and Treatment Algorithms Study Using HPV Testing in Africa.” https://clinicaltrials.gov/ show/NCT04794660 [Internet]. 2021; Available from: https://www.cochranelibrary.com/central/doi/10.1002/central/CN02269209/full |
| 1. Nicolau AIO, Lima TM, Vasconcelos CTM, Carvalho FHC, Aquino P de S, Pinheiro AKB. Telephone interventions in adherence to receiving the pap test report: A randomized clinical trial. Rev Lat Am Enfermagem. 2017;25. |
| 1. RBR-3w3vnc. Effects of telephone interventions on adherence to receive the results of examination of prevention of cervical cancer. http://www.who.int/ trialsearch/-Trial2.aspx?TrialID=RBR-3w3vnc [Internet]. 2016; Available from: https://www.cochranelibrary.com/central/doi/10.1002/central/CN-01841405/full |
| 1. Walsh J PMSROEGGDN, Green L, Walsh J, Potter M, Salazar R, Ozer E, et al. PreView: a Randomized Trial of a Multi-site Intervention in Diverse Primary Care to Increase Rates of Age-Appropriate Cancer Screening. JGIM J Gen Intern Med [Internet]. 2020 Feb;35(2):449–56. Available from: https://search.ebscohost.com/login.aspx?direct=true&db=ccm&AN=141726533&site=ehost-live |
| 1. Wearn A, Shepherd L. The impact of emotion-based mass media campaigns on stigma toward cervical screening non participation. J Appl Soc Psychol. 2020 May;50(5):289–98. |
| **Studies excluded due to duplication** |
| 1. Abadi MMM, Vakilian K, Safari V. Motivational interview on having pap test among middle-aged women – A counseling service in primary care. Fam Med Prim Care Rev. 2018;20(2):101–5. |
| 1. Abdullah F, Su TT. Applying the Transtheoretical Model to evaluate the effect of a call-recall program in enhancing Pap smear practice: A cluster randomized trial. Prev Med (Baltim) [Internet]. 2013;57(SUPPL):S83–6. Available from: http://dx.doi.org/10.1016/j.ypmed.2013.02.001 |
| 1. Okeke EN, Adepiti CA, Ajenifuja KO. What is the price of prevention? New evidence from a field experiment. J Health Econ [Internet]. 2013;32(1):207–18. Available from: http://dx.doi.org/10.1016/j.jhealeco.2012.10.001 |

**Supplementary Table S4: List of studies ongoing and awaiting classification**

| **Ongoing studies** |
| --- |
| 1. Arrossi S, Paolino M, Orellana L, Thouyaret L, Kohler RE, Viswanath K. Mixed-methods approach to evaluate an mHealth intervention to increase adherence to triage of human papillomavirus-positive women who have performed self-collection (the ATICA study): study protocol for a hybrid type i cluster randomized effectiveness-imp. Trials [Internet]. 2019;20(1 CC-Gynaecological, Neuro-oncology and Orphan Cancer). Available from: https://www.cochranelibrary.com/central/doi/10.1002/central/CN-01915662/full |
| 1. ChiCTR1800015671. A community health worker-led multimedia intervention to increase cervical cancer screening uptake among South Asians women: a randomized controlled trial. http://www.who.int/trialsearch/Trial2.aspx?-TrialID=ChiCTR1800015671 [Internet]. 2018; Available from: https://www.cochranelibrary.com/central/doi/10.1002/central/CN-01898819/full |
| 1. ChiCTR1800017897. Community-based educational intervention for cervical cancer prevention (CEDIC trial) in Nepal: a cluster randomized controlled trial. http://www.who.int/trialsearch/Trial2.aspx?TrialID=ChiCTR1800017897 [Internet]. 2018; Available from: https://www.cochranelibrary.com/central/ doi/10.1002/central/CN-01908009/full |
| 1. IRCT20120215009014N. Effect of group counseling based on protection-motivation theory versus no counseling on cervical cancer screening in postmenopausal women. http://www.who.int/trialsearch/Trial2.aspx?TrialID-=IRCT20120215009014N251 [Internet]. 2018; Available from: https://www.cochranelibrary.com/central/doi/10.1002/central/CN-01951047/full |
| 1. IRCT2014010516071N. The impact of training for Pap smears to detect early cervical cancer. http://www.who.int/trialsearch/Trial2.aspx?TrialID= IRCT2014-010516071N1 [Internet]. 2014; Available from: https://www.cochranelibrary.com/central/doi/10.1002/central/CN-01801270/full |
| 1. IRCT2015051215015N. The effect of training by health volunteers on performance of Pap smear test among women under their coverage. http://www.who.int/trialsearch/-Trial2.aspx?TrialID=IRCT2015051215015N6 [Internet]. 2015; Available from: https://www.cochranelibrary.com/central/doi/ 10.1002/central/CN-01892348/full |
| 1. IRCT2015090123852N. A Comparison of SMS and face to face training impacts on performance health screening tests in middle-aged women in Ahvaz. http://www.who.int/trialsearch/Trial2.aspx?TrialID=IRCT2015090123852N1 [Internet]. 2015; Available from: https://www.cochranelibrary.com/central/ doi/10.1002/central/CN-01880883/fullHenry Kitchener |
| 1. IRCT2016062928702N. The impact of training to do pap smear for early dignosis of cervical cancer. http://www.who.int/trialsearch/Trial2.aspx? TrialID=IRCT2016-0629287-02N1 [Internet]. 2016; Available from: https://www.cochranelibrary.com/central/doi/10.1002/central/CN-01891429/full |
| 1. IRCT20191206045626N1. Design, Implementation and Evaluation of a Cervical Cancer Prevention Behavior Training Program for Women of Reproductive Age. http://www.who.int/trialsearch/Trial2.aspx?TrialID= IRCT20191206045626N1 [Internet]. 2020; Available from: https://www.cochranelibrary.com/central/doi/ 10.1002/central/CN-02171121/full |
| 1. IRCT20200408046998N1. Evaluate the effect of Health Education on Knowledge, Attitude and Perception Regarding Cervical Cancer and its Screening among Women of Rural Areas of Punjab. http://www.who.int/ trialsearch/ Trial2.aspx?TrialID=IRCT2020-0408046998N1 [Internet]. 2020; Available from: https://www.cochranelibrary.com/-central/doi/10.1002/central/CN-02239511/full |
| 1. ISRCTN12767014. Integrated cervical cancer screening in Mayuge district Uganda (ASPIRE Mayuge). http://www.who.int/trialsearch/Trial2.aspx? TrialID=ISRCTN1276-7014 [Internet]. 2019; Available from: https://www.cochranelibrary.com/central/doi/10.1002/central/CN-01972651/full |
| 1. ISRCTN15608265. Cervical screening self-test study. http://www.who.int/-trialsearch/Trial2.aspx?TrialID=ISRCTN15608265 [Internet]. 2017; Available from: https://www.cochranelibrary.com/central/doi/10.1002/central/  CN-01894143/full |
| 1. Nakisige C, Trawin J, Mitchell-Foster S, Payne BA, Rawat A, Mithani N, et al. Integrated cervical cancer screening in Mayuge District Uganda (ASPIRE Mayuge): a pragmatic sequential cluster randomized trial protocol. BMC Public Health [Internet]. 2020;20(1). |
| 1. NCT02509702. Use of SMSs to Improve Attendance to Cervical Cancer Follow-up Screening. https://clinicaltrials.gov/show/NCT02509702 [Internet]. 2015; Available from: https://www.cochranelibrary.com/central/doi/10.1002/ central/CN-01491214/full |
| 1. NCT03076879. Increasing Participation in Cervical Cancer Screening and Risk for Beliefs/Attitudes Among Women at Risk. https://clinicaltrials.gov/show/ NCT03076879 [Internet]. 2017; Available from: https://www.cochranelibrary.com/ central/doi/-10.1002/central/CN-01577408/full |
| 1. NCT03281135. Optimizing Cervical Cancer Screening Modalities. https://clinicaltrials.gov/show/NCT03281135 [Internet]. 2017; Available from: https://www.cochranelibrary.com/central/doi/10.1002/central/CN-01592741/full |
| 1. NCT03514459. Testing an Implementation Science Tool to Increase Cervical Cancer Screening in Mombasa, Kenya. https://clinicaltrials.gov/-show/ NCT03514459 [Internet]. 2018; Available from: https://www.cochranelibrary.com/ central/doi/10.1002/central/CN-01599427/full |
| 1. NCT03540069. Reducing Barriers and Sustaining Utilization of a Cervical Cancer Screening Program in Rural Senegal. https://clinicaltrials.gov-/show/ NCT03540069 [Internet]. 2018; Available from: https://www.cochranelibrary.com/ central/doi/10.1002/central/CN-01659926/full |
| 1. NCT03808064. Community-based Intervention for Cervical Cancer Screening Uptake in Nepal. https://clinicaltrials.gov/show/NCT03808064 [Internet]. 2019; Available from: https://www.cochranelibrary.com/central/doi/10.1002/central/ CN-01702068/full |
| 1. NCT04000503. Integrated Cervical Cancer Screening in Mayuge District Uganda (ASPIRE Mayuge). https://clinicaltrials.gov/show/NCT04000503 [Internet]. 2019; Available from: https://www.cochranelibrary.com/central/ doi/10.1002/central/CN-01952859/full |
| 1. NCT04286243. Prevention of Cervical Cancer Through an HPV-based Screen-and-treat Strategy in Malawi. https://clinicaltrials.gov/show/NCT04286243 [Internet]. 2020; Available from: https://www.cochranelibrary.com/central/ doi/10.1002/central/CN-02088554/full |
| 1. NCT04307433. Storytelling Intervention to Promote Cervical Cancer Screening Uptakes Among Malawian Women Living With Human Immunodeficiency Virus. https://clinicaltrials.gov/show/NCT04307433 [Internet]. 2020; Available from: https://www.cochranelibrary.com/central/doi/10.1002/central/CN-02089054/full |
| 1. NCT04756440. The Effect of the Program on the Awareness of Roma Women on Cervical Cancer. https://clinicaltrials.gov/show/NCT04756440 [Internet]. 2021; Available from: https://www.cochranelibrary.com/central/doi/10.1002/ central/CN-02235237/full |
| 1. NCT04960748. Promoting Cervical Cancer Screening Through the Advocacy of Screened Women. https://clinicaltrials.gov/show/NCT04960748 [Internet]. 2021; Available from: https://www.cochranelibrar.com/central/doi/10.1002/ central/CN-02290356/full |
| 1. PACTR201305000526384. The use of the mobile phone short text message service to enhance cervical cancer screening at Thika Hospital, Kiambu County, Kenya(Smartscreening). http://www.who.int/trialsearch/Trial2.aspx?TrialID= PACTR201305000526384 [Internet]. 2013; Available from: https://www.cochranelibrary.com/central/doi/10.1002/central/CN-01840002/full |
| 1. PACTR201808126223676. The demand of cervical cancer screening among adult women in Tigray region, Ethiopia. http://www.who.int/trialsearch/ Trial2.aspx?TrialID=PACTR201808126223676 [Internet]. 2018; Available from: https://www.cochranelibrary.com/central/doi/10.1002/central/CN-01907015/full |
| 1. PACTR202003570419141. The Effect of Health Education Intervention rendered by Community Health Workers to Community Women in Promoting Cervical Cancer Screening Awareness and Behavior in Tanzania. http://www.who.int/trialsearch/Trial2.aspx?TrialID=PACTR202003570419141 [Internet]. 2020; Available from: https://www.cochranelibrary.com/central/ doi/10.1002/central/CN-02173364/full |
| 1. RBR-93ykhs. Behavioral and educational intervention: effects on return visit rates to receive the results of the Pap smear. http://www.who.int/trialsearch/ Trial2.aspx?-TrialID=RBR-93ykhs [Internet]. 2015; Available from: https://www.cochranelibrary.com/central/doi/10.1002/central/CN-01823570/full |
| 1. SLCTR/2015/015. A study to assess the effectiveness of selected health educational methods to improve cervical screening in a population of women in the Puttalam district. http://www.who.int/trialsearch/Trial2.aspx?TrialID= SLCTR/2015/015 [Internet]. 2015; Available from: https://www.cochranelibrary.com/central/doi/10.1002/central/CN-01878037/full |
| 1. Wong CL, Choi KC, Law BMH, Chan DNS, So WKW. Effects of a Community Health Worker-Led Multimedia Intervention on the Uptake of Cervical Cancer Screening among South Asian Women: A Pilot Randomized Controlled Trial. Int J Environ Res Public Health [Internet]. 2019;16(17). Available from: https://res.mdpi.com/d_attachment-/ijerph/ijerph-16-03072/article_deploy/ijerph-16-03072-v3.pdf |
| **Studies awaiting classifiaction** |
| 1. Jentschke M KJ, Hillemanns P. Hasco study protocol: pilot study for systematic HPV self-sampling for non-responders to the cervical cancer screening program. Oncol Res Treat. 2020;43:29‐30. |
| 1. TCTR20200205001. Effectiveness of Educational Intervention and WhatApp follow-up to improve Pap smear uptake among post-natal women in Seremban, Negeri Sembilan. http://www.who.int/trialsearch/Trial2.aspx?TrialID= TCTR20200205001 [Internet]. 2020; Available from: https://www.cochranelibrary.com/central/doi/10.1002/central/CN-02189578/full |
| 1. Antinyan A, Bertoni M, Corazzini L. Social Science & Medicine Cervical cancer screening invitations in low and middle income countries : Evidence from Armenia. Social Science & Medicine. 2021;273(January). |

**Supplementary Table S5: PRISMA checklist**

| **Section and Topic** | **Item #** | **Checklist item** | **Location where item is reported** |
| --- | --- | --- | --- |
| **TITLE** | | |  |
| Title | 1 | Identify the report as a systematic review. | Pg.1 |
| **ABSTRACT** | | |  |
| Abstract | 2 | See the PRISMA 2020 for Abstracts checklist. | Pg.1 |
| **INTRODUCTION** | | |  |
| Rationale | 3 | Describe the rationale for the review in the context of existing knowledge. | Pg.2-3 |
| Objectives | 4 | Provide an explicit statement of the objective(s) or question(s) the review addresses. | Pg.3 |
| **METHODS** | | |  |
| Eligibility criteria | 5 | Specify the inclusion and exclusion criteria for the review and how studies were grouped for the syntheses. | Pg.3 |
| Information sources | 6 | Specify all databases, registers, websites, organizations, reference lists and other sources searched or consulted to identify studies. Specify the date when each source was last searched or consulted. | Pg.3 |
| Search strategy | 7 | Present the full search strategies for all databases, registers and websites, including any filters and limits used. | Supplementary table 1 |
| Selection process | 8 | Specify the methods used to decide whether a study met the inclusion criteria of the review, including how many reviewers screened each record and each report retrieved, whether they worked independently, and if applicable, details of automation tools used in the process. | Pg.3 |
| Data collection process | 9 | Specify the methods used to collect data from reports, including how many reviewers collected data from each report, whether they worked independently, any processes for obtaining or confirming data from study investigators, and if applicable, details of automation tools used in the process. | Pg.3 |
| Data items | 10a | List and define all outcomes for which data were sought. Specify whether all results that were compatible with each outcome domain in each study were sought (e.g. for all measures, time points, analyses), and if not, the methods used to decide which results to collect. | Pg.3-4 |
|  | 10b | List and define all other variables for which data were sought (e.g. participant and intervention characteristics, funding sources). Describe any assumptions made about any missing or unclear information. | Pg.3-4 |
| Study risk of bias assessment | 11 | Specify the methods used to assess risk of bias in the included studies, including details of the tool(s) used, how many reviewers assessed each study and whether they worked independently, and if applicable, details of automation tools used in the process. | Pg.4 |

| **Section and Topic** | **Item #** | **Checklist item** | **Location where item is reported** |
| --- | --- | --- | --- |
| Effect measures | 12 | Specify for each outcome the effect measure(s) (e.g. risk ratio, mean difference) used in the synthesis or presentation of results. | Pg.4 |
| Synthesis methods | 13a | Describe the processes used to decide which studies were eligible for each synthesis (e.g. tabulating the study intervention characteristics and comparing against the planned groups for each synthesis (item #5)). | Pg.4 |
|  | 13b | Describe any methods required to prepare the data for presentation or synthesis, such as handling of missing summary statistics, or data conversions. | Pg.4 |
|  | 13c | Describe any methods used to tabulate or visually display results of individual studies and syntheses. | Pg.4 |
|  | 13d | Describe any methods used to synthesize results and provide a rationale for the choice(s). If meta-analysis was performed, describe the model(s), method(s) to identify the presence and extent of statistical heterogeneity, and software package(s) used. | Pg.4 |
|  | 13e | Describe any methods used to explore possible causes of heterogeneity among study results (e.g. subgroup analysis, meta-regression). | Pg.4 |
|  | 13f | Describe any sensitivity analyses conducted to assess robustness of the synthesized results. | Pg.4 |
| Reporting bias assessment | 14 | Describe any methods used to assess risk of bias due to missing results in a synthesis (arising from reporting biases). | Pg.4 |
| Certainty assessment | 15 | Describe any methods used to assess certainty (or confidence) in the body of evidence for an outcome. | Pg.4-5 |
| **RESULTS** | | |  |
| Study selection | 16a | Describe the results of the search and selection process, from the number of records identified in the search to the number of studies included in the review, ideally using a flow diagram. | Pg.5 |
|  | 16b | Cite studies that might appear to meet the inclusion criteria, but which were excluded, and explain why they were excluded. | Pg.5 |
| Study characteristics | 17 | Cite each included study and present its characteristics. | Pg.5-6 |
| Risk of bias in studies | 18 | Present assessments of risk of bias for each included study. | Pg.6 |
| Results of individual studies | 19 | For all outcomes, present, for each study: (a) summary statistics for each group (where appropriate) and (b) an effect estimate and its precision (e.g. confidence/credible interval), ideally using structured tables or plots. | Table 1 |
| Results of syntheses | 20a | For each synthesis, briefly summarize the characteristics and risk of bias among contributing studies. | Figure 2 and 6 |
| **Section and Topic** | **Item #** | **Checklist item** | **Location where item is reported** |
| Results of syntheses (cont.) | 20b | Present results of all statistical syntheses conducted. If meta-analysis was done, present for each the summary estimate and its precision (e.g. confidence/credible interval) and measures of statistical heterogeneity. If comparing groups, describe the direction of the effect. | Pg.16-21 |
|  | 20c | Present results of all investigations of possible causes of heterogeneity among study results. | Pg.10-13 |
|  | 20d | Present results of all sensitivity analyses conducted to assess the robustness of the synthesized results. | Pg.14 |
| Reporting biases | 21 | Present assessments of risk of bias due to missing results (arising from reporting biases) for each synthesis assessed. | Pg.11 |
| Certainty of evidence | 22 | Present assessments of certainty (or confidence) in the body of evidence for each outcome assessed. | Figure 6 |
| **DISCUSSION** | | |  |
| Discussion | 23a | Provide a general interpretation of the results in the context of other evidence. | Pg.14 |
|  | 23b | Discuss any limitations of the evidence included in the review. | Pg.14 |
|  | 23c | Discuss any limitations of the review processes used. | Pg.15 |
|  | 23d | Discuss implications of the results for practice, policy, and future research. | Pg.14 |
| **OTHER INFORMATION** | | |  |
| Registration and protocol | 24a | Provide registration information for the review, including register name and registration number, or state that the review was not registered. | Pg.3 |
|  | 24b | Indicate where the review protocol can be accessed, or state that a protocol was not prepared. | Pg.3 |
|  | 24c | Describe and explain any amendments to information provided at registration or in the protocol. | Supplementary 1 |
| Support | 25 | Describe sources of financial or non-financial support for the review, and the role of the funders or sponsors in the review. | Pg.17 |
| Competing interests | 26 | Declare any competing interests of review authors. | Pg.17 |
| Availability of data, code and other materials | 27 | Report which of the following are publicly available and where they can be found: template data collection forms; data extracted from included studies; data used for all analyses; analytic code; any other materials used in the review. | Pg.17 |

**Supplementary Table S6: Included studies by types of interventions**

| 1. Counseling/Health Education |
| --- |
| 1. Abadi 2018 (Iran) |
| 1. Dinshaw 2008 (India) |
| 1. Koc 2019 (Turkey) |
| 1. Kurt 2019 (Turkey) |
| 1. Mittra 2010 (India) |
| 1. Risi, 2004 (South Africa) |
| 1. Rosser 2015 (Kenya) |
| 1. Sossauer 2014 (Cameroon) |
| 1. Romli, 2020 (Malaysia) |
| 1. Atinel, 2020 (Turkey) |
| 1. Samami, 2021 (Iran) |
| 1. Abu, 2020 (Ethiopia) |
| 1. Shokoohi, 2020 (Iran) |
| 1. Reminding/Invitation |
| 1. Rashild, 2014 (Malaysia) |
| 1. Abdullah 2013 (Malaysia) |
| 1. Messaging |
| 1. Adonis 2016 (South Africa) |
| 1. Erwin 2019 (Tanzania) |
| 1. Okundade 2021 (Nigeria) |
| 1. Procedure (Community based self-sampling HPV vs Hospital based HPV or VIA) |
| 1. Gizaw 2019 (Ethiopia) |
| 1. Huchko 2018 (Western Kenya) |
| 1. Mitchell 2015 (Uganda) |
| 1. Modibbo 2017 (Nigeria) |
| 1. Moses 2015 (Uganda) |
| 1. Subsidized cost |
| 1. Okeke 2016 (Nigeria) |

**Supplementary Table S7: Judgement for risk of bias assessment using the Cochrane Risk of Bias Tool for Randomized Controlled Trials**

| **Bias** | **Authors’ judgement** | **Support for judgement** |
| --- | --- | --- |
| **Abadi 2018** | | |
| Random sequence generation (selection bias) | Low risk | “the participants were placed in two groups (control and intervention), each with 45 subjects, using randomized block design in two blocks (A and B).” |
| Allocation concealment (selection bias) | Unclear risk | Not mentioned |
| Blinding of participants and personnel (performance bias) | Unclear risk | Not mentioned |
| Blinding of outcome assessment (detection bias) | Unclear risk | Not mentioned |
| Incomplete outcome data (attrition bias) | Low risk | Of these, two dropped out and were replaced by two individuals selected through random selection. No loss to follow up. |
| Selective reporting (reporting bias) | Low risk | It is clear that all pre-specified and expected outcomes of interest are reported. |
| Other bias | Low risk | No |
| **Abdullah 2013** | | |
| Random sequence generation (selection bias) | Low risk | “Using a computer generated simple randomization method”. |
| Allocation concealment (selection bias) | Unclear risk | “Randomization was revealed after recruitment of the final school to ensure concealment of allocation.” |
| Blinding of participants and personnel (performance bias) | Low risk | “The study was a two armed, parallel group, unblinded, cluster randomized controlled trial” however there is not specified influenced on the outcomes. |
| Blinding of outcome assessment (detection bias) | Low risk | “The study was a two armed, parallel group, unblinded, cluster randomized controlled trial” however there is not specified influenced on the outcomes. |
| Incomplete outcome data (attrition bias) | Low risk | Two participants from the intervention arm and three from the control arm were lost to follow up, leaving 398 women for analysis (n= 199 participants in each arm). |
| Selective reporting (reporting bias) | Low risk | It is clear that all pre-specified and expected outcomes of interest are reported. They also stated about the missing data during the study period. |
| Other bias | Low risk | There is no other bias. |
| Recruitment bias | Low risk | Using a computer-generated simple randomization method in SPSSv15, each of the 40 public secondary schools which had agreed to participate were randomized into either the intervention (n=20) or control (n=20) groups whereby all teachers from the same school (cluster) were assigned to the same group. |
| Baseline imbalance | Low risk | No difference in baseline characteristics. |
| Loss of clusters | Low risk | No evidence of lost of clusters. |
| Incorrect analysis | High risk | No cluster correction. |
| Comparability with individually randomized trials | Low risk | Analyze with individually |
| **Abu 2020** | | |
| Random sequence generation (selection bias) | Unclear risk | The eight health centers were randomized into intervention and control arms, four health centers each. |
| Allocation concealment (selection bias) | Unclear risk | not mentioned |
| Blinding of participants and personnel (performance bias) | Low risk | not mentioned, less likely to influence |
| Blinding of outcome assessment (detection bias) | Low risk | not mentioned, less likely to influence |
| Incomplete outcome data (attrition bias) | Low risk | In the follow-up data collection, two thousand one hundred forty participants (89.2%) have participated. |
| Selective reporting (reporting bias) | Low risk | No |
| Other bias | Low risk | No |
| Recruitment bias | Unclear risk | 14 health centers were providing cervical cancer screening in Addis Ababa, all using VIA. Eight high test load health centers were selected based on the number of clients in the past year. |
| Baseline imbalance | Low risk | No |
| Loss of clusters | Low risk | No |
| Incorrect analysis | High risk | No cluster correction |
| Comparability with individually randomized trials | Low risk | Not different |
| **Adonis 2016** | | |
| Random sequence generation (selection bias) | Unclear risk | “A structured email was sent to eligible females in August 2013 and then again 3 months later in November 2013. Females randomly received a loss-, gain-, or neutral-framed email.” |
| Allocation concealment (selection bias) | Unclear risk | “A structured email was sent to eligible females in August 2013 and then again 3 months later in November 2013. Females randomly received a loss-, gain-, or neutral-framed email.” |
| Blinding of participants and personnel (performance bias) | Unclear risk | Not mentioned. |
| Blinding of outcome assessment (detection bias) | Unclear risk | Data were collected from the health insurance database using Current Procedural Terminology (CPT) codes. |
| Incomplete outcome data (attrition bias) | Low risk | Total 42 were lost to follow up; 5.29% (n=19) in control, 8.33% (n=12) in gained, 9.1% (n=11) in loss framed; which is less than 20%. |
| Selective reporting (reporting bias) | Low risk | It is clear that all pre-specified and expected outcomes of interest are reported. They also stated about the missing data during the study period. |
| Other bias | Low risk | No |
| **Atinel 2020** | | |
| Random sequence generation (selection bias) | Low risk | Computer generated random number table |
| Allocation concealment (selection bias) | Unclear risk | Not mentioned |
| Blinding of participants and personnel (performance bias) | Low risk | Unblinded, less likely to influence |
| Blinding of outcome assessment (detection bias) | Low risk | Blinding to data collectors, statistical analysis and report writing |
| Incomplete outcome data (attrition bias) | Unclear risk | Not mentioned |
| Selective reporting (reporting bias) | Unclear risk | Not mentioned |
| Other bias | Unclear risk | No |
| **Dinshaw 2008** | | |
| Random sequence generation (selection bias) | Unclear risk | Not mentioned |
| Allocation concealment (selection bias) | Unclear risk | Not mentioned |
| Blinding of participants and personnel (performance bias) | Low risk | “both arms are provided with colours coded project identity cards.” Physicians are blinded to the patient group (both arms) |
| Blinding of outcome assessment (detection bias) | Low risk | Not stated, less likely to influence on outcome |
| Incomplete outcome data (attrition bias) | Low risk | less than 20% |
| Selective reporting (reporting bias) | High risk | Only show the results of intervention group. And, only compliance rate is reported. Outcome of interest can't be found. |
| Other bias | High risk | The study did not mention data on control arms. Not cited the previous study which describing the study design. Tables, there was no mentioned about N? not described the intervention vs control arms. The data are combined for both breast and cervix screening. It is hard to visualized for each category. |
| Recruitment bias | Unclear risk | They only mentioned the randomization of the clusters into IG and CG but did not mention the recruitment of participants. |
| Baseline imbalance | High risk | Not mentioned about the control group. |
| Loss of clusters | Low risk | No |
| Incorrect analysis | High risk | No cluster correction |
| Comparability with individually randomized trials | Low risk | No |
| **Erwin 2019** | | |
| Random sequence generation (selection bias) | Low risk | “use multistage systematic stratified random sampling. Unique study identifiers (USIDs) were generated by Medic Mobile to implement randomization.” Randomization is sorted in excel. |
| Allocation concealment (selection bias) | Unclear risk | "Fieldworkers responsible for recruitment remained unaware of allocations so that blinding was maintained. "But do not mentioned details. |
| Blinding of participants and personnel (performance bias) | Low risk | Fieldworkers responsible for recruitment remained unaware of allocations so that blinding was maintained. |
| Blinding of outcome assessment (detection bias) | Low risk | The primary outcome: the uptake of CC screening within 60 days recorded by a field worker. |
| Incomplete outcome data (attrition bias) | Low risk | There were 15 participants were excluded from SMS+eVoucher group due to insufficient follow-up period. |
| Selective reporting (reporting bias) | Low risk | No |
| Other bias | Low risk | No |
| **Gizaw 2019** | | |
| Random sequence generation (selection bias) | Low risk | The randomization list was created by using a unique allocation ID. The randomization was conducted using Research Randomizer Software. |
| Allocation concealment (selection bias) | Unclear risk | Not mentioned clearly. |
| Blinding of participants and personnel (performance bias) | Low risk | Not stated, less likely to influence the outcome. |
| Blinding of outcome assessment (detection bias) | Low risk | Not stated, less likely to influence the outcome. |
| Incomplete outcome data (attrition bias) | Low risk | No |
| Selective reporting (reporting bias) | Low risk | No |
| Other bias | Low risk |  |
| Recruitment bias | Low risk | The study mentioned the randomization process of clusters. |
| Baseline imbalance | High risk | Among 11 clusters in each arm, about one third of the participants in the VIA arm reported that travelling to hospital was difficult, while very few participants reported similarly in the HPV arm. |
| Loss of clusters | Low risk | No |
| Incorrect analysis | High risk | No cluster correction. |
| Comparability with individually randomized trials | Low risk | Not different. |
| **Huchko 2018** | | |
| Random sequence generation (selection bias) | Low risk | "12 communities were randomized 1:1 using an allocation sequence generated by Stata/MP version 11". |
| Allocation concealment (selection bias) | Unclear risk | Not mentioned |
| Blinding of participants and personnel (performance bias) | Low risk | Not stated, less likely to influence the outcome (uptake) |
| Blinding of outcome assessment (detection bias) | Unclear risk | Not stated, less likely to influence the outcome (uptake) |
| Incomplete outcome data (attrition bias) | Low risk | No |
| Selective reporting (reporting bias) | Low risk | No |
| Other bias | Low risk | No |
| **Koc 2019** | | |
| Random sequence generation (selection bias) | Low risk | random number table |
| Allocation concealment (selection bias) | Unclear risk | not mentioned |
| Blinding of participants and personnel (performance bias) | Unclear risk | not mentioned |
| Blinding of outcome assessment (detection bias) | Unclear risk | not mentioned |
| Incomplete outcome data (attrition bias) | Low risk | No |
| Selective reporting (reporting bias) | Low risk | No |
| Other bias | Low risk | No |
| **Kurt 2019** | | |
| Random sequence generation (selection bias) | Low risk | Used block randomization to be ordered and numbered the participants. |
| Allocation concealment (selection bias) | Unclear risk | Not mentioned. |
| Blinding of participants and personnel (performance bias) | Low risk | Not stated, less likely to influence the outcome (uptake) |
| Blinding of outcome assessment (detection bias) | Low risk | Not stated, less likely to influence the outcome (uptake) |
| Incomplete outcome data (attrition bias) | Low risk | No |
| Selective reporting (reporting bias) | Low risk | No |
| Other bias | Low risk | No |
| **Mitchell 2015** | | |
| Random sequence generation (selection bias) | Unclear risk | The participants were randomly selected but the study did not mention the method |
| Allocation concealment (selection bias) | Unclear risk | Oral presentation did not mention for this domain. |
| Blinding of participants and personnel (performance bias) | Unclear risk | Oral presentation did not mention for this domain. |
| Blinding of outcome assessment (detection bias) | Unclear risk | Oral presentation did not mention for this domain. |
| Incomplete outcome data (attrition bias) | Unclear risk | Oral presentation did not mention for this domain. |
| Selective reporting (reporting bias) | Unclear risk | Oral presentation did not mention for this domain. |
| Other bias | Unclear risk | Oral presentation did not mention for this domain. |
| **Mittra 2010** | | |
| Random sequence generation (selection bias) | Unclear risk | These 20 clusters were then randomly assigned to the screening and control (health education). Not mentioned how to generate random sequence. |
| Allocation concealment (selection bias) | Unclear risk | Not mentioned |
| Blinding of participants and personnel (performance bias) | Low risk | Unblinded, less likely to be influenced. |
| Blinding of outcome assessment (detection bias) | Unclear risk | Unblinded, less likely to be influenced. |
| Incomplete outcome data (attrition bias) | Low risk | Less than 20% |
| Selective reporting (reporting bias) | Low risk | No |
| Other bias | Low risk | No |
| Recruitment bias | Low risk | Twenty slum clusters were selected by single stage, simple random sampling technique. |
| Baseline imbalance | Low risk | No |
| Loss of clusters | Low risk | No |
| Incorrect analysis | Unclear risk | The ‘‘Intracluster Correlation’’ and ‘‘Design Effect’’ was calculated using MLWIN software. Analysis is on intention-to-treat basis. |
| Comparability with individually randomized trials | Low risk | No |
| **Modibbo 2017** | | |
| Random sequence generation (selection bias) | High risk | We assigned the 200 odd numbers on the list to the self-sampling group and the 200 even numbers to the hospital-based sampling group. |
| Allocation concealment (selection bias) | Unclear risk | Not mentioned |
| Blinding of participants and personnel (performance bias) | Low risk | Unblinded (no blinding but measurement unlikely to be influenced) |
| Blinding of outcome assessment (detection bias) | Low risk | Unblinded (no blinding but measurement unlikely to be influenced) |
| Incomplete outcome data (attrition bias) | Unclear risk | Not mentioned |
| Selective reporting (reporting bias) | Low risk | No |
| Other bias | Low risk | No |
| **Moses 2015** | | |
| Random sequence generation (selection bias) | Low risk | Simple randomisation, a randomisation list was generated using SAS 9.3 |
| Allocation concealment (selection bias) | Low risk | Study ID and allocation were recorded on cards, which were concealed in an envelope and kept in a locked cabinet in the research office. Study ID and allocation were recorded on cards, which were concealed in an envelope and kept in a locked cabinet in the research office. |
| Blinding of participants and personnel (performance bias) | Low risk | Unblined (no blinding but measurement unlikely to be influenced) |
| Blinding of outcome assessment (detection bias) | Low risk | Unblined (no blinding but measurement unlikely to be influenced) |
| Incomplete outcome data (attrition bias) | Low risk | No attrition |
| Selective reporting (reporting bias) | Low risk | No |
| Other bias | Low risk | No |
| **Okeke 2016** | | |
| Random sequence generation (selection bias) | Low risk | Women chose scratch cards with three covered circles. |
| Allocation concealment (selection bias) | Low risk | A scratch card with three covered circles numbered 1–3. |
| Blinding of participants and personnel (performance bias) | Low risk | Unblined (no blinding but measurement unlikely to be influenced) |
| Blinding of outcome assessment (detection bias) | Low risk | Unblined (no blinding but measurement unlikely to be influenced) |
| Incomplete outcome data (attrition bias) | Unclear risk | Not mentioned |
| Selective reporting (reporting bias) | Low risk | No |
| Other bias | Low risk | No |
| **Okunade 2016** | | |
| Random sequence generation (selection bias) | Low risk | Enrolled women were randomly assigned to either the text message (mHealth) arm or the standard of care (usual care) arm using a 1:1 random assignment sequence generated by the study statistician from the Random Allocation software version 1.0 (May 2004). |
| Allocation concealment (selection bias) | Low risk | The allocation sequence was kept in sealed opaque envelopes that were stored in locked file cabinets at the study sites until participants’ assignments were completed. |
| Blinding of participants and personnel (performance bias) | Low risk | Unblinded, but less likely to influence |
| Blinding of outcome assessment (detection bias) | Low risk | Unblinded, but less likely to influence |
| Incomplete outcome data (attrition bias) | Low risk | No |
| Selective reporting (reporting bias) | Low risk | No |
| Other bias | Low risk | No |
| **Rashid 2014** | | |
| Random sequence generation (selection bias) | Low risk | One thousand women had been randomly selected by computer-generated number and 250 women were then randomly assigned to the four different methods of recall. |
| Allocation concealment (selection bias) | Unclear risk | Not mentioned |
| Blinding of participants and personnel (performance bias) | Low risk | Unblinded, less likely to influence |
| Blinding of outcome assessment (detection bias) | Low risk | Unblinded, less likely to influence |
| Incomplete outcome data (attrition bias) | High risk | Attrition is more than 20%. |
| Selective reporting (reporting bias) | Low risk | No |
| Other bias | Low risk | No |
| **Risi 2004** | | |
| Random sequence generation (selection bias) | Unclear risk | Seventy-five census areas were randomly sampled from the remaining 451 (16.6%). Ten households per census area were randomly selected and one eligible woman identified from the census. |
| Allocation concealment (selection bias) | Low risk | with sealed envelope. |
| Blinding of participants and personnel (performance bias) | High risk | Unblinded |
| Blinding of outcome assessment (detection bias) | High risk | Unblinded |
| Incomplete outcome data (attrition bias) | Low risk | No |
| Selective reporting (reporting bias) | Low risk | No |
| Other bias | Unclear risk | There were two interventions and second is non-randomized; assess outcome after second intervention. |
| **Romli 2020** | | |
| Random sequence generation (selection bias) | Unclear risk | The districts were randomly assigned as the intervention group and control group. Alor Setar district was assigned as the intervention group and Sungai Petani as the control group. |
| Allocation concealment (selection bias) | Unclear risk | Not mentioned |
| Blinding of participants and personnel (performance bias) | Unclear risk | Not mentioned |
| Blinding of outcome assessment (detection bias) | Unclear risk | Not mentioned |
| Incomplete outcome data (attrition bias) | Low risk | No |
| Selective reporting (reporting bias) | Low risk | No |
| Other bias | Low risk | No |
| Recruitment bias | Unclear risk | Not mentioned clearly |
| Baseline imbalance | Low risk | No |
| Loss of clusters | Low risk | No |
| Incorrect analysis | High risk | No cluster correction |
| Comparability with individually randomized trials | Low risk | Not different |
| **Rooser 2015** | | |
| Random sequence generation (selection bias) | Low risk | Randomization was done in eight blocks. |
| Allocation concealment (selection bias) | Unclear risk | Not mentioned |
| Blinding of participants and personnel (performance bias) | Low risk | Unblined (no blinding but measurement unlikely to be influenced) |
| Blinding of outcome assessment (detection bias) | Low risk | Unblined (no blinding but measurement unlikely to be influenced) |
| Incomplete outcome data (attrition bias) | High risk | 78% completed; attrition is more than 20%. |
| Selective reporting (reporting bias) | Low risk | No |
| Other bias | Low risk | No |
| **Samami 2011** | | |
| Random sequence generation (selection bias) | Low risk | with random number table |
| Allocation concealment (selection bias) | Unclear risk | Not mentioned. |
| Blinding of participants and personnel (performance bias) | Unclear risk | Not mentioned |
| Blinding of outcome assessment (detection bias) | Unclear risk | Not mentioned |
| Incomplete outcome data (attrition bias) | Low risk | No |
| Selective reporting (reporting bias) | Low risk | No |
| Other bias | Low risk | No |
| Recruitment bias | Unclear risk | Not mentioned clearly |
| Baseline imbalance | Low risk | No |
| Loss of clusters | Low risk | No |
| Incorrect analysis | High risk | No cluster correction |
| Comparability with individually randomized trials | Low risk | Not different |
| **Shokoohi 2020** | | |
| Random sequence generation (selection bias) | Unclear risk | We randomly invited subjects to come in during one of the experimental weeks, independently of treatment group allocation. |
| Allocation concealment (selection bias) | Unclear risk | Not mentioned |
| Blinding of participants and personnel (performance bias) | Unclear risk | Not mentioned |
| Blinding of outcome assessment (detection bias) | Unclear risk | Not mentioned |
| Incomplete outcome data (attrition bias) | Low risk | No |
| Selective reporting (reporting bias) | Low risk | No |
| Other bias | Low risk | No |
| Recruitment bias | High risk | Samples have entered the groups using convenience sampling. |
| Baseline imbalance | Low risk | No |
| Loss of clusters | Low risk | No |
| Incorrect analysis | High risk | No cluster correction |
| Comparability with individually randomized trials | Low risk | Not different |
| **Sossauer 2014** | | |
| Random sequence generation (selection bias) | High risk | Participants were randomized in groups of 10 by order of arrival alternating into intervention or control group. |
| Allocation concealment (selection bias) | High risk | No |
| Blinding of participants and personnel (performance bias) | High risk | Unblinded |
| Blinding of outcome assessment (detection bias) | High risk | Unblinded |
| Incomplete outcome data (attrition bias) | Low risk | No |
| Selective reporting (reporting bias) | Low risk | No |
| Other bias | Low risk | No |

**Supplementary Table S8: Summary of reported outcomes of the included studies**

| ID | Author, year | Outcomes | | | | | | | |
| --- | --- | --- | --- | --- | --- | --- | --- | --- | --- |
|  |  | **Uptake of cervical cancer screening** | **Willingness to get screening**  **(categorical)** | **Satisfactory Knowledge** | **Knowledge score** | **Attitude score** | **Willingness to get screening**  **(continuous)** | **Cost effectiveness** | **Total** |
| 1 | Abadi, 2018*** | / | / |  | / (high SD) | / (high SD) |  |  | **4** |
| 2 | Abdullah, 2013 | / |  |  |  |  |  |  | **1** |
| 3 | Abu, 2020 | / |  |  |  |  |  |  | **1** |
| 4 | Adonis, 2016 | / |  |  |  |  |  |  | **1** |
| 5 | Altinel, 2020** | / (% change) |  |  |  |  |  |  | **1** |
| 6 | Dinshaw, 2008** | / (only int arm) |  |  |  |  |  |  | **1** |
| 7 | Erwin, 2019 | / |  |  |  |  |  |  | **1** |
| 8 | Gizaw, 2019 | / |  |  |  |  |  |  | **1** |
| 9 | Huchko, 2018* | / |  |  |  |  |  | / (no SD) | **2** |
| 10 | Koc, 2019**** |  |  |  |  |  | / (median) |  | **1** |
| 11 | Kurt, 2019 | / |  |  | / |  | / |  | **3** |
| 12 | Mitchell, 2015 | / |  |  |  |  |  |  | **1** |
| 13 | Mittra, 2010** | / (only int arm) | / (only int arm) |  |  |  |  |  | **2** |
| 14 | Modibbo, 2017 | / |  |  |  |  |  |  | **1** |
| 15 | Moses, 2015* | / |  |  |  |  |  | / (only int arm) | **2** |
| 16 | Okeke, 2016 | / (%) |  |  |  |  |  |  | **1** |
| 17 | Okunade, 2021 | / |  |  |  |  |  |  | **1** |
| 18 | Rashid, 2014 | / |  |  |  |  |  | / | **2** |
| 19 | Risi, 2004 | / |  |  |  |  |  |  | **1** |
| 20 | Romli, 2020 | / (%) |  |  | / | / (negative) |  |  | **3** |
| 21 | Rosser, 2015 | / | / |  | / |  |  |  | **3** |
| 22 | Samami, 2021 | / |  |  | / |  |  |  | **2** |
| 23 | Shokoohi, 2020 |  |  |  |  |  |  |  |  |
| 24 | Sossauer, 2014 |  | / | / |  |  |  |  | **2** |
|  | **Total** | **21** | **4** | **1** | **5** | **2** | **2** | **3** | **38** |

Int arm, intervention arm; SD, standard deviation; negative, mentioned as negative attitude which means lower score was the better attitude

Note: For the outcomes of **social stigma, stress, anxiety, favorable attitude, satisfaction and satisfaction score**, there was not measured. There was no outcome of interest in Shokoohi, 2020.

*Studies that have additional report for another outcome (cost effective) in addition to main study report for primary outcome (uptake screening). However, one study did not show SD and one study measured intervention arm only; thus, they are not included in data analysis.

**Study did not mention the control arm (Dinshaw 2008); Study that mentioned the outcome of uptake of screening and willingness to get screening for intervention arm only while the outcome of the control arm was the participation in health education (Mittra 2010).; Study mentioned % of change in uptake but no actual number (Atinel 2020); thus, it was not included in data analysis.

***Study that have very low Mean and high SD for knowledge score and attitude score; thus, it was not included in data analysis.

**** Study that mentioned with median was not included in data analysis

**Supplementary Table S9:** Summary of findings table (single intervention compared with control for uptake cervical cancer screening)

| **Certainty assessment** | | | | | | | **№ of patients** | | **Effect** | | **Certainty** | **Importance** |
| --- | --- | --- | --- | --- | --- | --- | --- | --- | --- | --- | --- | --- |
| **№ of studies** | **Study design** | **Risk of bias** | **Inconsistency** | **Indirectness** | **Imprecision** | **Other considerations** | **Single intervention** | **standard/routine care/no intervention** | **Relative (95% CI)** | **Absolute (95% CI)** |  |  |
| **Uptake of cervical cancer screening** | | | | | | | | | | | | |
| 13 | randomised trials | serious^a^ | serious^b^ | not serious | not serious | none | 1253/2847 (44.0%) | 705/2455 (28.7%) | **RR 1.47** (1.19 to 1.82) | **135 more per 1,000** (from 55 more to 235 more) | ⨁⨁◯◯ Low |  |
| **Willingness to get cervical cancer screening** | | | | | | | | | | | | |
| 3 | randomised trials | very serious^c^ | serious^d^ | not serious | serious^e^ | none | 335/358 (93.6%) | 301/361 (83.4%) | **RR 1.18** (0.96 to 1.45) | **150 more per 1,000** (from 33 fewer to 375 more) | ⨁◯◯◯ Very low |  |
| **Knowledge score** | | | | | | | | | | | | |
| 3 | randomised trials | serious^f^ | serious^g^ | not serious | not serious | none | 286 | 292 | - | SMD **0.73 SD higher** (0.08 higher to 1.38 higher) | ⨁⨁◯◯ Low |  |

a. We downgraded one level for serious risk of bias due to unclear information for selection bias

b. We downgraded one level for serious inconsistency due to substantial statistical heterogeneity (I^2^=88%).

c. We downgraded two levels for very serious risk of bias due to high risk for selection bias in one study and unclear information for allocation concealment in two studies

d. We downgraded one level for serious inconsistency due to substantial statistical heterogeneity (I^2^=93%).

e. We downgraded one level for serious imprecision due to wide confidence interval (RRR=15%)

f. We downgraded one level for serious risk of bias due to unclear information for selection bias

g. We downgraded one level for serious inconsistency due to substantial statistical heterogeneity (I^2^=91%).

**Supplementary Table S10:** Summary of findings table (single intervention compared with other intervention for uptake cervical cancer screening)

| **Certainty assessment** | | | | | | | **№ of patients** | | **Effect** | | **Certainty** | **Importance** |
| --- | --- | --- | --- | --- | --- | --- | --- | --- | --- | --- | --- | --- |
| **№ of studies** | **Study design** | **Risk of bias** | **Inconsistency** | **Indirectness** | **Imprecision** | **Other considerations** | **Single intervention** | **Other intervention** | **Relative (95% CI)** | **Absolute (95% CI)** |  |  |
| **Uptake of cervical cancer screening (brochure vs invitation)** | | | | | | | | | | | | |
| 1 | randomised trials | serious^a^ | not serious | not serious | very serious^b^ | none | 38/119 (31.9%) | 30/119 (25.2%) | **RR 1.27** (0.84 to 1.90) | **68 more per 1,000** (from 40 fewer to 227 more) | ⨁◯◯◯ Very low |  |
| **Uptake of cervical cancer screening (Reminding with phone call vs registered letter)** | | | | | | | | | | | | |
| 1 | randomised trials | serious^c^ | not serious | not serious | serious^d^ | none | 86/250 (34.4%) | 50/250 (20.0%) | **RR 1.72** (1.27 to 2.32) | **144 more per 1,000** (from 54 more to 264 more) | ⨁⨁◯◯ Low |  |
| **Uptake of cervical cancer screening (Reminding with Phone call vs SMS)** | | | | | | | | | | | | |
| 1 | randomised trials | serious^e^ | not serious | not serious | serious^f^ | none | 86/250 (34.4%) | 54/250 (21.6%) | **RR 1.59** (1.19 to 2.13) | **127 more per 1,000** (from 41 more to 244 more) | ⨁⨁◯◯ Low |  |
| **Uptake of cervical cancer screening (Reminding with Registered letter vs SMS)** | | | | | | | | | | | | |
| 1 | randomised trials | serious^g^ | not serious | not serious | very serious^h^ | none | 50/250 (20.0%) | 54/250 (21.6%) | **RR 0.93** (0.66 to 1.30) | **15 fewer per 1,000** (from 73 fewer to 65 more) | ⨁◯◯◯ Very low |  |
| **Uptake of cervical cancer screening (Messaging with loss-framed vs gained framed)** | | | | | | | | | | | | |
| 1 | randomised trials | serious^i^ | not serious | not serious | very serious^j^ | none | 16/176 (9.1%) | 11/175 (6.3%) | **RR 1.45** (0.69 to 3.03) | **28 more per 1,000** (from 19 fewer to 128 more) | ⨁◯◯◯ Very low |  |
| **Uptake of cervical cancer screening (Messaging with 15 SMS vs one SMS)** | | | | | | | | | | | | |
| 1 | randomised trials | serious^k^ | not serious | not serious | serious^l^ | none | 35/298 (11.7%) | 12/281 (4.3%) | **RR 2.75** (1.46 to 5.19) | **75 more per 1,000** (from 20 more to 179 more) | ⨁⨁◯◯ Low |  |
| **Uptake of cervical cancer screening (Subsidized cost N0 vs N50)** | | | | | | | | | | | | |
| 1 | randomised trials | not serious | not serious | not serious | very serious^m^ | none | 65/362 (18.0%) | 53/351 (15.1%) | **RR 1.19** (0.85 to 1.66) | **29 more per 1,000** (from 23 fewer to 100 more) | ⨁⨁◯◯ Low |  |
| **Uptake of cervical cancer screening (Subsidized cost N0 vs N100)** | | | | | | | | | | | | |
| 1 | randomised trials | not serious | not serious | not serious | very serious^n^ | none | 65/362 (18.0%) | 37/330 (11.2%) | **RR 1.60** (1.10 to 2.33) | **67 more per 1,000** (from 11 more to 149 more) | ⨁⨁◯◯ Low |  |
| **Uptake of cervical cancer screening (Subsidized cost N50 vs N100)** | | | | | | | | | | | | |
| 1 | randomised trials | not serious | not serious | not serious | very serious^o^ | none | 53/351 (15.1%) | 37/330 (11.2%) | **RR 1.35** (0.91 to 1.99) | **39 more per 1,000** (from 10 fewer to 111 more) | ⨁⨁◯◯ Low |  |
| **Uptake of cervical cancer screening (Community based HPV vs Hospital based HPV)** | | | | | | | | | | | | |
| 2 | randomised trials | serious^p^ | not serious | not serious | serious^q^ | none | 365/374 (97.6%) | 234/409 (57.2%) | **RR 1.67** (1.53 to 1.82) | **383 more per 1,000** (from 303 more to 469 more) | ⨁⨁◯◯ Low |  |
| **Willingness to get cervical cancer screening (brochure vs invitation)** | | | | | | | | | | | | |
| 1 | randomised trials | serious^r^ | not serious | not serious | serious^s^ | none | 119 | 119 | - | MD **0.11 higher** (0.06 lower to 0.28 higher) | ⨁⨁◯◯ Low |  |
| **Knowledge score (brochure vs invitation)** | | | | | | | | | | | | |
| 1 | randomised trials | serious^t^ | not serious | not serious | serious^u^ | none | 119 | 119 | - | MD **1.78 higher** (1.2 higher to 2.36 higher) | ⨁⨁◯◯ Low |  |

a. We downgraded one level for serious risk of bias due to unclear information for allocation concealment

b. We downgraded two levels for very serious imprecision due to wide confidence interval (RRR=15%), and few events (<400).

c. We downgraded one level for serious risk of bias due to unclear information for allocation concealment.

d. We downgraded one level for serious imprecision due to few events (<400).

e. We downgraded one level for serious risk of bias due to unclear information for allocation concealment.

f. We downgraded one level for serious imprecision due to few events (<400).

g. We downgraded one level for serious risk of bias due to unclear information for allocation concealment

h. We downgraded two levels for very serious imprecision due to wide confidence interval (RRR=15%), and few events (<400).

i. We downgraded one level for serious risk of bias due to unclear information for allocation concealment

j. We downgraded two levels for very serious imprecision due to wide confidence interval (RRR=15%), and few events (<400).

k. We downgraded one level for serious risk of bias due to unclear information for allocation concealment.

l. We downgraded one level for serious imprecision due to few events (<400).

m. We downgraded two levels for very serious imprecision due to wide confidence interval (RRR=15%), and few events (<400).

n. We downgraded two levels for very serious imprecision due to wide confidence interval (RRR=15%) and few events (<400).

o. We downgraded two levels for very serious imprecision due to wide confidence interval (RRR=15%), and few events (<400).

p. We downgraded one level for serious risk of bias due to unclear information for allocation concealment and high risk of bias in random sequence generation in one study but it

contributes lower weight.

q. We downgraded one level for serious imprecision due to few events (<400).

r. We downgraded one level for serious risk of bias due to unclear information for allocation concealment.

s. We downgraded one level for serious imprecision due to small sample size (<400).

t. We downgraded one level for serious risk of bias due to unclear information for allocation concealment.

u. We downgraded one level for serious imprecision due to small sample size (<400).

**Supplementary Table S11:** Summary of findings table (combined interventions compared with single intervention for uptake cervical cancer screening)

| **Certainty assessment** | | | | | | | **№ of patients** | | **Effect** | | **Certainty** | **Importance** |
| --- | --- | --- | --- | --- | --- | --- | --- | --- | --- | --- | --- | --- |
| **№ of studies** | **Study design** | **Risk of bias** | **Inconsistency** | **Indirectness** | **Imprecision** | **Other considerations** | **Combined interventions** | **Single intervention** | **Relative (95% CI)** | **Absolute (95% CI)** |  |  |
| **Uptake of cervical cancer screening** | | | | | | | | | | | | |
| 2 | randomised trials | serious^a^ | serious^b^ | not serious | serious^c^ | none | 120/390 (30.8%) | 115/817 (14.1%) | **RR 2.20** (1.54 to 3.14) | **169 more per 1,000** (from 76 more to 301 more) | ⨁◯◯◯ Very low |  |
| **Willingness to get cervical cancer screening** | | | | | | | | | | | | |
| 1 | randomised trials | serious^d^ | not serious | not serious | serious^e^ | none | 118 | 238 | - | MD **0.07 higher** (0.06 lower to 0.2 higher) | ⨁⨁◯◯ Low |  |
| **Knowledge score** | | | | | | | | | | | | |
| 1 | randomised trials | serious^f^ | serious^g^ | not serious | very serious^h^ | none | 118 | 238 | - | MD **0.89 higher** (0.85 lower to 2.64 higher) | ⨁◯◯◯ Very low |  |

a. We downgraded one level for serious risk of bias due to unclear information for selection bias.

b. We downgraded one level for serious inconsistency due to substantial statistical heterogeneity (I^2^=61%).

c. We downgraded one level for serious imprecision due to few events (<400).

d. We downgraded one level for serious risk of bias due to unclear information for selection bias.

e. We downgraded one level for serious imprecision due to small sample size (<400).

f. We downgraded one level for serious risk of bias due to unclear information for selection bias.

g. We downgraded one level for serious inconsistency due to substantial statistical heterogeneity (I^2^=96%).

h. We downgraded two levels for very serious imprecision due to wide confidence interval (MID at 2), and small sample size (<400).
